# Differential effects of CMV infection on the viability of cardiac cells

**DOI:** 10.1038/s41420-023-01408-y

**Published:** 2023-04-03

**Authors:** Santosh K. Yadav, Flobater I. Gawargi, Mohammad H. Hasan, Ritesh Tandon, Jason W. Upton, Paras K. Mishra

**Affiliations:** 1 Department of Cellular and Integrative Physiology, University of Nebraska Medical Center, Omaha, NE USA; 2 Department of Cell and Molecular Biology, Center for Immunology and Microbial Research, University of Mississippi Medical Center, Jackson, MS USA; 3 Department of Medicine, University of Mississippi Medical Center, Jackson, MS USA; 4 Department of Biological Sciences, Auburn University, Alabama, AL USA

**Keywords:** Diseases, Physiology

## Abstract

Cytomegalovirus (CMV) is a widely prevalent herpesvirus that reaches seroprevalence rates of up to 95% in several parts of the world. The majority of CMV infections are asymptomatic, albeit they have severe detrimental effects on immunocompromised individuals. Congenital CMV infection is a leading cause of developmental abnormalities in the USA. CMV infection is a significant risk factor for cardiovascular diseases in individuals of all ages. Like other herpesviruses, CMV regulates cell death for its replication and establishes and maintains a latent state in the host. Although CMV-mediated regulation of cell death is reported by several groups, it is unknown how CMV infection affects necroptosis and apoptosis in cardiac cells. Here, we infected primary cardiomyocytes, the contractile cells in the heart, and primary cardiac fibroblasts with wild-type and cell-death suppressor deficient mutant CMVs to determine how CMV regulates necroptosis and apoptosis in cardiac cells. Our results reveal that CMV infection prevents TNF-induced necroptosis in cardiomyocytes; however, the opposite phenotype is observed in cardiac fibroblasts. CMV infection also suppresses inflammation, reactive oxygen species (ROS) generation, and apoptosis in cardiomyocytes. Furthermore, CMV infection improves mitochondrial biogenesis and viability in cardiomyocytes. We conclude that CMV infection differentially affects the viability of cardiac cells.

## Introduction

Cytomegalovirus (CMV) is a double-stranded DNA virus that belongs to the beta-herpesviridae family [1]. The serological prevalence of CMV infection ranges from 40–95% of the population worldwide, and approximately 1/3^rd^ of children in the USA are infected with CMV by the age of five [2–4]. CMV infection is subclinical in immunocompetent individuals whereas reactivation of the virus in immunocompromised individuals leads to multi-organ disease and death [5, 6]. In vivo studies on cell tropism have suggested that endothelial, epithelial, fibroblast, and smooth muscle cells are the major targets of CMV lytic infection, whereas myeloid cells of hematopoietic origin including monocytes and polymorphonuclear leukocytes are permissive to infection but do not support lytic replication [7–10]. CMV infection is linked to an increased risk of cardiovascular disease (CVD), and a meta-analysis of community-based prospective studies showed that CMV infection attributes to 13.4% of CVD incidence [11]. CMV infection is involved in allograft rejection in pediatric heart transplant recipients [12]. Also, CMV infection mismatch in donor and recipient patients causes complications impairing outcomes in heart transplantation [13]. The inflammatory status of chronic heart failure (CHF) patients is directly related to CMV infection. CMV infection promotes the production of pro-inflammatory cytokines, and the concentrations of cytokines (tumor necrosis factor-alpha (TNFα), Interleukin-6 (IL-6), IL-17A, and IL-1β) are positively correlated to the production of antibodies against CMV in CHF patients [14]. Cytokines are released from the dying cardiac cells, and they induce adverse cardiac remodeling [15]. Long-term CMV infection is linked to the instigation of adverse cardiac remodeling by inducing cardiac hypertrophy and fibrosis [16]. However, the direct effect of CMV infection on cardiac cell death remains unclear.

Human CMV (HCMV) and mouse CMV (MCMV) have similar pathogenesis on the heart, and both promote inflammation to induce myocarditis [16–18]. In the present study, we have used MCMV, and determined its effects on cardiac cell death. At least six forms of cell death have been reported in the heart, namely apoptosis, necroptosis, mitochondrial-mediated necrosis, pyroptosis, ferroptosis, and autophagic cell death [19]. In the present study, we focused on apoptosis and necroptosis cell death mechanisms in cardiomyocytes and fibroblast cells. MCMV replicates and establishes latency in host cells by synthesizing viral suppressors of necroptosis and apoptosis signaling molecules [20–22]. Apoptosis is a well-characterized caspase-dependent cell death mechanism, which is triggered by both caspase-9-mediated intrinsic (mitochondrial-dependent) and caspase-8-mediated extrinsic signaling pathways [23, 24]. Extrinsic cell death signaling involves the activation of different death receptors, including TNF receptor-1 (TNFR1) [25]. In the presence of a caspase-8 inhibitor, extrinsic signaling can shift from apoptosis to a form of programmed necrosis termed necroptosis [26]. Necroptotic cell death is mediated by the interaction of Receptor Interacting Protein Kinase-3 (RIPK3) with adaptor proteins, such as RIPK1, Z-DNA binding protein-1 (ZBP1, also termed DAI) - a virus-induced nucleic acid sensor protein, and TIR-domain-containing adaptor-inducing interferon-β (TRIF), which is involved in response to activation of Toll-Like Receptors (TLRs) [27–29]. These interactions occur through a small protein-protein interaction motif known as RIP homotypic interaction motif (RHIM). Activation of different receptors and interaction of adapter proteins with RIPK3 lead to RIPK3 phosphorylation and activation [30]. The activated RIPK3 phosphorylates Mixed Lineage Kinase-Like (MLKL), a downstream effector molecule in the necroptosis pathway, which undergoes oligomerization and subsequently cell membrane insertion leading to cell swelling, disruption of plasma membrane integrity, and cell lysis [31, 32].

MCMV encodes the M45 gene, which is the first identified Viral Inhibitor of Receptor- interacting protein Activation (vIRA) that inhibits necroptosis [29]. M45 contains a RHIM and inhibits RIPK3 through RHIM-dependent interaction during infection to suppress the ZBP1‐RIPK3‐dependent necroptosis [33]. Increased necroptosis is an important innate anti-viral response by host cells [34]. Furthermore, MCMV suppresses apoptosis by encoding a Viral Inhibitor of Caspase-8-dependent apoptosis (vICA) - a product of the M36 gene, and loss of this gene product (∆M36) promotes caspase-8-mediated apoptosis [35–37]. Notably, caspase-8 is also involved in necroptosis and inflammatory signaling. Caspase-8 negatively regulates RIPK3 to inhibit necroptosis and restricts RIPK3-dependent proinflammatory IL-1β production [38, 39]. MCMV also employs two inhibitors of intrinsic apoptosis, the Viral Mitochondria-localized Inhibitor of Apoptosis (vMIA) encoded by m38.5, and the Viral Inhibitor of Bak Oligomerization (vIBO) encoded by m41.1, which suppresses the pro-apoptotic Bcl2-family members Bax and Bak, respectively. The loss of m38.5 and m41.1 gene products leads to apoptosis during MCMV infection [40–43]. Although MCMV infection is reported to inhibit non-cardiac cell death, how MCMV infection affects necroptosis and apoptosis in cardiac cells remains unclear. Here, we used primary cardiomyocytes and cardiac fibroblasts to investigate MCMV infection-induced regulation of necroptosis and apoptosis.

## Results

### MCMV infection prevents TNFα-induced necroptosis in cardiomyocytes possibly by suppressing inflammation

**Fig. 1:**
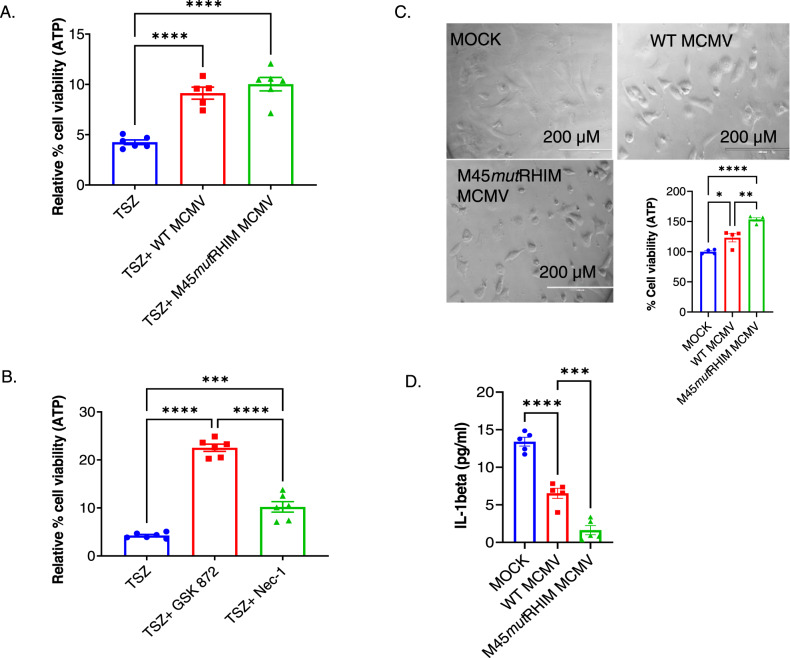
MCMV infection prevents necroptosis in cardiomyocytes independent of ZBP1-RIPK3 pathway by suppressing upstream inflammation. **A** Viability of neonatal cardiomyocytes obtained from C57BL/6 J mice were treated with necroptosis inducing agents TSZ in the presence and absence of MCMV (WT or M45*mut*RHIM mutant MCMV) infection. **B** Viability of neonatal cardiomyocytes treated with TSZ in the presence and absence of necroptosis inhibitor GSK872 (RIPK3 inhibitor) or Nec-1 (RIPK1 inhibitor). **C** Phase-contrast image and viability assessed by ATP levels in WT and M45*mut*RHIM mutant CMV-infected of neonatal cardiomyocytes. **D** Inflammatory cytokine Interleukin-1 beta (IL-1β) was measured in the culture medium of neonatal cardiomyocytes infected with WT or M45*mut*RHIM mutant MCMV. WT CMV = K-181-bac. One-way ANOVA followed by Tukey’s multiple comparison test was performed. Values are mean ± SE. Each point represents one sample. *n* = 4–6. ***P < 0.001; ****P < 0.0001.

To determine the effect of MCMV infection on necroptosis in cardiomyocytes, we standardized the multiplicity and duration of MCMV infection using HL1 cell line, which has murine origin and exhibits several features of cardiomyocytes [44]. After time course and dose optimization studies (Fig. S1), we decided to use 5 MOI of MCMV and 24 h treatment duration for all studies with cardiac cells. We infected primary (neonatal) cardiomyocytes with WT MCMV (K181-bac) in the presence of necroptosis inducer TSZ, where T stands for TNFα, S stands for smac-mimetic compound SM-164, and Z stands for a pan-caspase inhibitor zVAD [45]. Since infection of susceptible cells with a virus mutated in the RHIM domain of M45 (M45*mut*RHIM) induces ZBP1-RIPK3-dependent necroptosis [33], we also infected primary cardiomyocytes with M45*mut*RHIM MCMV, treated them with TSZ, and evaluated their cell viability. We found that MCMV infection prevents TSZ-induced necroptosis in cardiomyocytes, which is independent of the DAI/ZBP1-RIPK3-mediated necroptosis pathway (Fig. 1A).

To determine the role of RIPK3 and RIPK1 in necroptosis of cardiomyocytes (without MCMV infection), we treated primary cardiomyocytes with GSK872 (a RIPK3 inhibitor) and Nec-1 (a RIPK1 inhibitor) in the presence of TSZ and evaluated cell viability. We observed that inhibition of both RIPK3 and RIPK1 blunts the detrimental effects of TSZ on cardiomyocyte viability (Fig. 1B). This finding demonstrates that the RIPK1 and RIPK3-mediated necroptosis contributes to decreased cell viability in cardiomyocytes. Furthermore, the RIPK3-mediated necroptosis has a more pronounced effect on cell viability than the RIPK1-mediated necroptosis in cardiomyocytes.

To evaluate if MCMV infection improves the viability of cardiomyocytes independent of necroptosis (in the absence of chemical inducer TSZ), we measured cell viability in uninfected (MOCK), and WT and M45*mut*RHIM MCMV infected primary cardiomyocytes. We observed that WT MCMV infection increased the viability of primary cardiomyocytes, which was further enhanced by M45*mut*RHIM mutation in MCMV (Fig. 1C). We also observed a similar effect of WT and M45*mut*RHIM CMV infection on the improvement of cell viability and attenuation of cytotoxicity in HL1 cells (Fig. S2). Altogether, these findings suggest that MCMV infection promotes the viability of cardiomyocytes irrespective of necroptosis, and that cardiomyocytes are resistant to MCMV-induced necroptosis.

To confirm that the improvement in cell viability of cardiomyocytes is mainly due to MCMV infection, we measured viral proteins required for successful MCMV infection (Immediate Early-1 or IE1, Early protein-1 or E1) and replication (MCMV envelope glycoprotein or gB) in uninfected (MOCK), and WT and M45*mut*RHIM MCMV-infected HL1 cells. We found that these three virus proteins were expressed to comparable levels in the WT and M45*mut*RHIM MCMV-infected HL1 cells and not in the uninfected cells (Fig. S3). These findings corroborate MCMV infection and demonstrate that improvement in the viability of HL1 cells was due to MCMV infection.

Inflammation is an upstream signal for necroptosis since inflammation induces TNFα, which in turn instigates necroptosis. In mice, MCMV infection in the heart promotes TNFα expression [18]. Furthermore, circulating levels of inflammatory cytokine IL-1β are positively associated with anti-HCMV titer in CHF patients [14]. Therefore, we measured the secreted IL-1β in the culture medium of uninfected, WT and M45*mut*RHIM MCMV-infected cardiomyocytes. We found a remarkable decrease in IL-1β in WT MCMV-infected cardiomyocytes, which was further decreased in M45*mut*RHIM MCMV-infected cardiomyocytes (Fig. 1D). These findings suggest that suppression of upstream inflammation could be a potential mechanism by which MCMV infection prevents initiation of necroptosis and presumably cell death in cardiomyocytes. In addition, our results suggest that the necroptosis-specific mutation (M45*mut*RHIM) in MCMV contributes to the attenuation of inflammation in cardiomyocytes.

### MCMV infection inhibits apoptosis in cardiomyocytes by reducing ROS

**Fig. 2:**
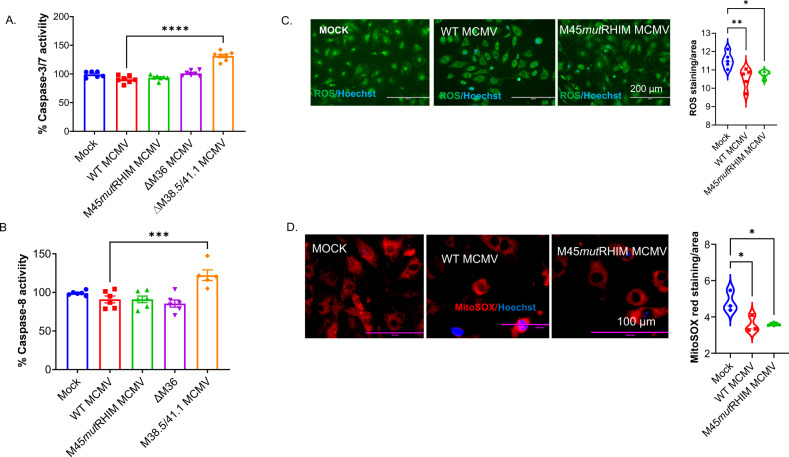
MCMV infection prevents apoptosis in cardiomyocytes by reducing ROS. **A**–**B**. Caspase-3/7 and caspase-8 activity in uninfected (MOCK), and WT, M45*mut*RHIM, ∆M36, and ∆M38.5/41.1 MCMV infected neonatal cardiomyocytes. **C** Staining of cellular oxidative stress (ROS) by CellROX and its quantification in neonatal cardiomyocytes infected with WT MCMV and M45*mut*RHIM MCMV. **D** Staining of mitochondrial ROS by MitoSOX and its quantification (*n* = 3 samples in each group). WT CMV = K-181-bac, ∆M36 = mutant CMV mutant promotes caspase-8-mediated apoptosis, ∆M38.5/41.1 CMV = mutant CMV promotes mitochondria-mediated (Bax/Bak) apoptosis. One-way ANOVA followed by Tukey’s multiple comparison test was performed. Values are mean ± SE. Each point represents one sample. *n* = 5–6 in (**A**–**B**) and *n* = 3 in (**C**–**D**). *P < 0.05; **P < 0.01; ***P < 0.001; ****P < 0.0001.

Apoptosis is triggered either by caspase-8 signaling that directly activates caspase-3/7 by cleaving pro-caspase-3/7 or by truncating Bcl-2 homology (BH)-3interacting-domain death agonist (BID) that induces mitochondrial-mediated caspase-3 activation through cytochrome c release [19]. We used two mutant MCMVs, one lacking caspase-8 inhibition (∆M36) and another lacking mitochondrial-mediated apoptosis inhibition (∆M38.5/41.1). To determine the effects of caspase-8 on cardiomyocyte viability, we infected primary cardiomyocytes with MOCK, WT, and three mutant MCMVs (M45*mut*RHIM, ∆M36, and ∆M38.5/41.1) and measured caspase-3/7 and caspase-8 activity. We found only mitochondrial-mediated apoptotic mutant MCMV (∆M38.5/41.1) infection was able to increase caspase-3/7 and caspase-8 activity (Fig. 2A, B). These findings suggest that MCMV infection inhibits mitochondrial-mediated apoptosis in cardiomyocytes independent of the necroptosis pathway.

Mitochondrial-mediated apoptosis is triggered by intrinsic stimuli such as reactive oxygen species (ROS). Notably, the necroptosis pathway may regulate ROS to influence mitochondrial-mediated apoptosis [46]. Thus, we measured cytosolic and mitochondrial ROS levels in MOCK, WT, and necroptosis mutant (M45*mut*RHIM) MCMV-infected primary cardiomyocytes. We observed that both WT and M45*mut*RHIM MCMV infections reduced the cytosolic and mitochondrial levels of ROS (Fig. 2C, D). Similar findings were also observed in HL1 cells (Fig. S4). These results suggest that CMV may inhibit mitochondrial-mediated apoptosis by decreasing ROS levels in cardiomyocytes.

### MCMV infection promotes mitochondrial biogenesis to increase the viability of cardiomyocytes

**Fig. 3:**
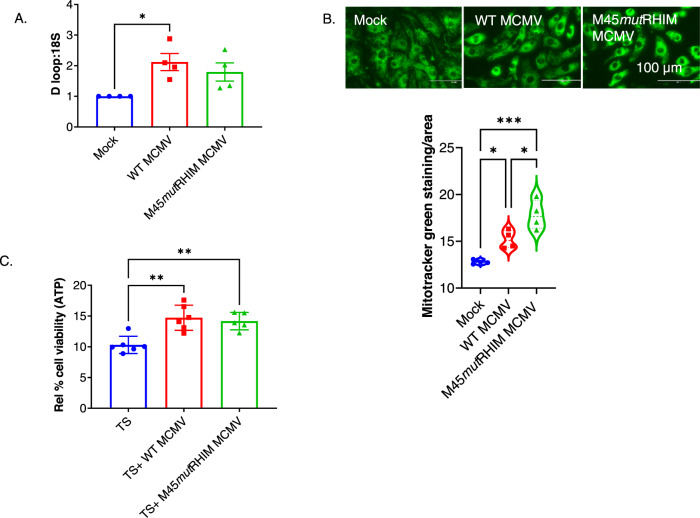
MCMV infection increases mitochondrial biogenesis to promote cell viability in primary cardiomyocytes. **A** Mitochondrial DNA copy number assessed by D-loop in uninfected (MOCK), WT MCMV, and M45mutRHIM MCMV infected neonatal cardiomyocytes. **B** Morphology and cell viability of uninfected and WT and M45mutRHIM MCMV infected neonatal primary cardiomyocytes. **C** Effect of apoptosis inducer TS on viability of uninfected, WT MCMV, and M45mutRHIM MCMV infected neonatal cardiomyocytes. WT CMV = K-181- bac. One-way ANOVA followed by Tukey’s multiple comparison test was performed. Values are mean ± SE. Each point represents one sample. *n* = 4–6 in each group. *P < 0.05; **P < 0.01; ***P < 0.001.

Impaired mitochondrial biogenesis may contribute to increases in ROS levels [47]. Thus, we measured mitochondria biogenesis (mitochondrial DNA copy number) and the number of mitochondria (using mitotracker green) in the uninfected (MOCK), WT MCMV, and M45*mut*RHIM MCMV-infected primary cardiomyocytes. Both WT and M45*mut*RHIM MCMV infection increased mitochondrial biogenesis and the number of mitochondria in cardiomyocytes (Fig. 3A, B). We also measured different mitochondrial biogenesis markers (PGC1α, TFAM, and mitotracker) in HL1 cells and observed increased mitochondrial biogenesis, complementing the results from primary cardiomyocytes (Fig. S5). The improved mitochondrial biogenesis may reduce ROS and subsequent ROS-induced apoptosis to improve cell viability. Thus, we determined cell viability in WT and M45*mut*RHIM MCMV-infected cardiomyocytes after 24 h treatment with apoptosis inducer TS, where T stands for TNFα and S stands for smac-mimetic compound SM-164. Both WT and M45*mut*RHIM MCMV infections blunt the detrimental effects of TS on cell viability in cardiomyocytes (Fig. 3C). These findings suggest that MCMV infection increased cell viability, potentially by improving mitochondrial biogenesis in cardiomyocytes.

### MCMV infection induces cell death in cardiac fibroblasts

**Fig. 4:**
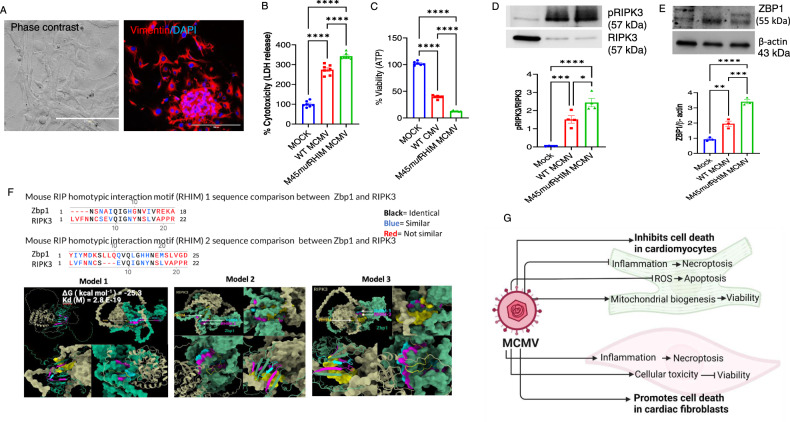
MCMV infection decreases cell viability by promoting necroptosis and apoptosis in primary cardiac fibroblasts. **A** Imaging cardiac fibroblasts immunofluorescence by Phase-contrast and vimentin. **B** Cell toxicity measurement by lactate dehydrogenase (LDH) release in uninfected (MOCK), WT MCMV, and M45*mut*RHIM MCMV infected cardiac fibroblasts. **C** Cell viability evaluation by ATP levels in uninfected (MOCK), WT MCMV, and M45*mut*RHIM MCMV infected cardiac fibroblasts. **D** Immunoblot and quantification of pRIPK3/RIPK3 in cardiac fibroblasts infected with WT and M45*mut*RHIM MCMV. **E** Immunoblot and quantification of ZBP1 in cardiac fibroblasts infected with WT and M45*mut*RHIM MCMV. **F** ZBP1 and RIP3 protein-protein-interactions in RHIM 1 and RHIM 2 domain. RHIM motif sequences for ZBP1 and RIPK3 were obtained from Uniprot database Q9QY24, Q9QZL0, respectively. Sequences were aligned using SnapGene software using global alignment tool (Needleman–Wunsch), identical amino acids are highlighted in black, similar amino acids in blue, and different amino acids in red. In 3 predicted models for ZBP1 binding to RIPK3, both ZBP1 and RIPK3 structures were obtained from alpha-fold database Q9QY24, Q9QZL0, respectively. Structural docking was performed using ClusPro for Protein-Protein docking. All models were analyzed for interaction sites, and only models with interactions through RHIM domains were considered in the analysis. Pymol and ChimeraX were used for analysis. Protein affinity binding was measured using prodigy webtool to calculate ∆G and Kd between the two proteins. **G** Schematic illustration of MCMV-mediated suppression of cell death in cardiomyocytes and induction of cell death in cardiac fibroblasts. BioRender was used to create this figure. WT CMV = K-181- bac. One-way ANOVA followed by Tukey’s multiple comparison test was performed. Values are mean ± SE. Each point represents one sample. *n* = 4–6 in each group. *P < 0.05; **P < 0.01; ***P < 0.001; ****P < 0.0001.

Since fibroblasts play a crucial role in cardiac fibrosis and dysfunction, we next evaluated how MCMV infection influences cardiac fibroblasts [48]. We first characterized cardiac fibroblasts isolated from the four to five-week-old murine heart and then evaluated their cell cytotoxicity and viability. The presence of vimentin, a marker of fibroblast, validated the isolation of uncontaminated primary cardiac fibroblasts (Fig. 4A). MCMV infection increased cytotoxicity (increased LDH levels) and impaired viability (decreased ATP levels) in cardiac fibroblasts (Fig. 4B, C). These results demonstrate that MCMV infection promotes cytotoxicity and decreases cell viability in primary cardiac fibroblasts. Moreover, the MCMV infection-induced cell death in cardiac fibroblasts was associated with an increase in TNFα levels (Fig. S6). Since TNFα promotes cell death via necroptosis, we evaluated necroptosis in cardiac fibroblasts after infection with WT and M45*mut*RHIM CMV. We observed that necroptosis markers (RIPK3 and ZBP1) were upregulated by WT MCMV infection and further enhanced by M45*mut*RHIM CMV infection in cardiac fibroblasts (Fig. 4D–E). Altogether, these findings demonstrate that MCMV infection promotes cell death via necroptosis in cardiac fibroblasts.

Notably, the molecular weight of ZBP1 varies between cardiomyocytes (42 kDa) and fibroblasts (55 kDa), pointing to a possibility of different ZBP1 variants in cardiomyocytes versus other cardiac cells (Figs. S7 and 4E). Our search in NCBI showed two validated splice variants (molecular weights 44 kDa and 20 kDa) of mouse ZBP1 where the second variant lacks both RHIMs (Fig. S8). ZBP1 interacts with RIPK3 via RHIM1 suggesting that splice variants without a RHIM (20 kDa) are unlikely to interact with RIPK3. ZBP1 is known to interact with several proteins, which are involved in inflammation and other cell signaling pathways, including the cell death pathway via RIPK1 and RIPK3 (Fig. S9). There is no defined or validated motif of ZBP1. To determine a predicted protein-protein interaction of ZBP1 and RIPK3, we acquired predicted ZBP1 protein structure using the AlphaFold protein structure database. We used both PyMOL and ChimeraX programs for predicted ZBP1 structural analysis and ClusPro web server for the predicted molecular docking of ZBP1 on RIPK3. Based on the high binding affinity analyzed by prodigy software, we propose three structural models for the predicted ZBP1 motif binding to RIPK3 through RHIM motifs, where each model represents a different docking position between the two proteins (Fig. 4F). However, these predicted models warrant further validation.

Altogether, these findings suggest that MCMV infection affects cell death differentially in cardiomyocytes and cardiac fibroblasts. In cardiomyocytes, MCMV infection promotes cell viability by inhibiting necroptosis and apoptosis, and by promoting mitochondrial biogenesis, whereas in cardiac fibroblasts, MCMV infection promotes cell death by inducing inflammation and necroptosis (Fig. 4G).

## Discussion

Several clinical studies have demonstrated that CMV infection promotes CVD; however, the impact of CMV infection on cardiac cell viability remains unclear. Our studies reveal that CMV infection differentially influences cardiac cell viability in the murine heart by promoting the viability of cardiomyocytes while decreasing the viability of cardiac fibroblasts. One of the potential mechanisms of improved viability in MCMV-infected cardiomyocytes is the inhibition of both necroptosis and apoptosis.

Host cells eliminate viruses by inducing programmed cell death, such as necroptosis. However, programming death of terminally differentiated cardiomyocytes is an irreparable loss that could lead to heart failure. Thus, limiting programmed cell death in cardiomyocytes at the expense of carrying out CMV infection is potentially a critical adaptive mechanism that prevents heart failure. Improving the viability of cardiomyocytes is also affordable to CMV infection, which could provide an opportunity for the virus to replicate in these cells. Thus, cardiomyocytes are in a unique position where it is advantageous for both the host and CMV to save the cell from death. In other cardiac cells, such as fibroblasts, which are not as indispensable as myocytes, virus-infected cells are programmed to die to eliminate virus infection. Thus, cardiomyocytes and other cardiac cells may have developed different strategies to adapt to virus infection. This is the first report that shows that the same virus differentially regulates the fate of two different cell types in the same tissue.

Herpesvirus is known to induce and block apoptosis at multiple steps during infection, and protect cells from the extrinsic cell death in a cell-type-dependent manner [49]. Our results show that MCMV infection promotes the viability of cardiomyocytes by increasing mitochondrial biogenesis, reducing ROS accumulation and inflammation, and inhibiting necroptotic and apoptotic signaling pathways. MCMV is known to use multiple strategies to evade the host cell’s programmed cell death mechanisms [50]. In cardiomyocytes, MCMV utilizes these established mechanisms to promote the survival of cardiomyocytes, although other unrecognized mechanisms may play additional roles. This is also the first report demonstrating that MCMV infection induces mitochondrial biogenesis and suppresses inflammation in cardiomyocytes.

DNA of CMV is detected in the atherosclerotic plaques, and CMV is involved in the development of atherosclerosis [51, 52]. However, there are inconsistent reports on the relationship of serum CMV seropositivity with the incidence of CVD [53, 54]. A recent meta-analysis on multiple studies that were performed to assess the effect of CMV infection on relative risk of CVD - ischemic heart disease, stroke, and cardiovascular death - revealed that CMV infection is associated with an increased risk of CVD [11]. However, none of these studies investigated the effect of CMV infection on cardiac cells. The present study reveals how MCMV infection differentially regulates cell viability in cardiomyocytes and cardiac fibroblasts, and their underlying molecular mechanisms. MCMV infection prevents the host cell death by modulating necroptosis [29]. In cardiomyocytes, both RIPK1 and RIPK3 inhibitors mitigate cytokine-induced cell death and thereby improve cell viability, which supports the previous report that necroptosis contributes to myocardial cell death [19]. MCMV requires the RHIM-containing vIRA to prevent the RIPK3-mediated necroptosis, and mutation in the RHIM (M45*mut*RHIM) augments TNFα-induced necroptosis [33]. In cardiomyocytes, M45*mut*RHIM does not sensitize cells to the TNFα-induced necroptosis, as cell viability after TSZ treatment remains unchanged between WT and M45*mut*RHIM MCMV-infected cardiomyocytes. Notably, MCMV infection decreases inflammation (IL-1β) in cardiomyocytes, which was enhanced by the M45*mut*RHIM mutation, pointing to a potential role of attenuated inflammation in the viability of cardiomyocytes. Based on these findings, we posit that the RHIM of MCMV vIRA may regulate inflammation but not TNFα-induced necroptosis in cardiomyocytes. How the RHIM regulates inflammation in cardiomyocytes remains unclear and requires further investigation.

RIPK3 domain needs adaptor protein ZBP1, a nucleic acid sensor, for necroptosis and IL-1β release [55]. We observed that IL-1β was decreased by the WT MCMV infection in cardiomyocytes, and the decrease was enhanced by the M45*mut*RHIM mutation. Thus, there is a possibility that RIPK3 and ZBP1 may have differential interactions in cardiomyocytes leading to the attenuation of inflammation and inhibition of necroptosis. ZBP1 also binds to the Z-nucleic acid-binding domain (ZBD) of the RNA-editing enzyme ADAR1, which is a negative regulator of ZBP1 [56]. We observed different molecular weights of ZBP1 in cardiomyocytes (42 kDa) and fibroblasts (55 kDa). At least two validated splice variants of mouse ZBP1 have been reported: (1) a full-length ZBP1 containing two RHIMs (RHIM1 and RHIM 2) and (2) a shorter variant with a predicted molecular weight of 20 kDa lacking any RHIMs. There is potentially more cell type or tissue-specific splice variants of mouse ZBP1, although that remains to be validated. Our observed 42 kDa splice variant could be a novel cardiomyocyte-specific splice variant, which warrants further studies. It is germane to report that human ZBP1 has more validated splice variants than mouse, and the human ZBP1 structure differs from the mouse ZBP1. Thus, the new mouse variants require further validation for their presence or absence in humans. Altogether, our findings suggest a possibility of a new cardiomyocyte-specific variant of ZBP1 (hereafter termed cZBP1), which is different from the cardiac fibroblast variant and contributes to an increase in the viability of cardiomyocytes after MCMV infection.

ZBP1 promotes IL-1β release via the RHIM-mediated interactions with RIPK1 [55]. Thus, there is a possibility that cZBP1 in cardiomyocytes may compromise IL-1β release causing a decrease in inflammation. We observed that attenuation of IL-1β level was comparatively higher in the M45*mut*RHIM MCMV-infected than the WT MCMV-infected cardiomyocytes. This points to a potential interaction of the MCMV vRIA with the cZBP1 to reduce IL-1β released during infection. As inflammation is an upstream regulator of necroptosis and potentially other forms of cell death, we posit that cZBP1 in cardiomyocytes reduces inflammation, downstream necroptosis, and possibly other cell death signaling resulting in increased viability after MCMV infection. Future studies focusing on interactions of cZBP1 with RIPK3 to regulate IL-1β are warranted.

Like all studies, ours has a few limitations. The first limitation is that we have used only in vitro models. Although we have used primary cardiomyocytes and cardiac fibroblasts in our studies to delineate the specific effects of MCMV infection on specific cardiac cells (cardiomyocytes versus cardiac fibroblasts), a cardiomyocyte-specific or fibroblast-specific loss and gain-of-function mouse model would provide a whole-body effect of MCMV infection. It could also delineate cardiac-specific versus the central mechanism-mediated effects of MCMV infection on the heart. The second limitation is the use of neonatal cardiomyocytes rather than the adult cardiomyocytes. This limitation is due to technical challenges in the culture and treatment of adult cardiomyocytes. We know that the gene expression of neonatal cardiomyocytes is not identical to that of the adult cardiomyocytes. However, despite these limitations, we have uncovered an unexpected difference in the way cardiomyocytes and cardiac fibroblasts respond to viral infection.

In summary, our results reveal that MCMV infection differentially regulates cell death in different cardiac cells. It promotes cell viability in cardiomyocytes while cell death in cardiac fibroblasts. We identify the potential regulatory mechanism by which MCMV infection increased cell viability in cardiomyocytes and cell death in cardiac fibroblasts. We predict a new splice variant of ZBP1 - cZBP1 - in cardiomyocytes, which potentially decreases inflammation and improves viability after MCMV infection. Furthermore, we demonstrate that necroptosis inhibition in cardiomyocytes was independent of RHIM-domain-dependent RIPK3 activation, and concomitant with reduced ROS and inflammation, and increased mitochondrial mass. In contrast to cardiomyocytes, MCMV infection promotes necroptosis and cellular toxicity to decrease viability in cardiac fibroblasts. These are novel and unique features of different cardiac cells to adapt to CMV infection.

## Materials and methods

### Generation of MCMV mutants

MCMV mutants M45*mut*RHIM, ∆M36, and ∆M38.5/41.1 have been previously described [29, 50, 57]. Wild type (K181-bac) and mutant viral stocks were generated, clarified, concentrated, and tittered by plaque assay as previously described using NIH 3T3 fibroblasts (ATCC CRL-1658) [58].

### Isolation and culture of neonatal cardiomyocytes

For Isolation of neonatal cardiomyocytes, one to two-day old pups from C57BL/6 J mice were decapitated and hearts were dissected out. Primary cardiomyocytes were isolated using Pierce Primary Cardiomyocyte Isolation Kit (Cat # 88281).

### Isolation and culture of neonatal cardiac fibroblast

Four to five-week-old C57BL/6 J mice hearts were extracted, washed in ice-cold PBS and HBSS, and minced in 1–3 mm^3^ pieces. The minced tissue was mixed with 0.1% collagenase II and incubated at 37 °C for an hour in a shaker incubator. Dulbecco’s Modified Eagle Medium (DMEM) medium was added to stop the enzymatic tissue lysis, and lysed tissue was washed in ice-cold HBSS. Cells were counted and cultured in complete DMEM medium containing 5 ng/ml fibroblast growth factor and used for further treatments.

### Viral infection

Primary neonatal cardiomyocytes, 0.5 × 10^6^ cells, were seeded in a 24-well plate at the time of isolation. After two days of culture, the cells were infected with the WT MCMV and the mutant MCMV at MOI = 5 for 24 h in the incomplete medium. After 24 h of incubation, the culture medium and cells were collected separately. Cell lysates were prepared by lysis of the cell pellet in the RIPA lysis buffer containing protease and phosphatase buffer. The culture medium was used for measuring Lactate Dehydrogenase (LDH) release and cytokine levels.

### Protein isolation and Western blotting

We used a standard method for Western blotting [59]. The primary antibodies used were pRIPK3 (Cat # 91702), RIPK3 (Cat # ab56164), ZBP1 (Cat # NBP1-76854), IE1 (anti-m123/IE1- clone IE1.01, Cat # R-MCMV-12), E1 (anti- M112-113/E1- clone- chroma-103, Cat # HR-MCMV-07), gB (anti- M55/gB- clone-M55.01, Cat # HR-MCMV-05), and β-actin (1:5000, ab56164). The secondary antibodies used were anti-rabbit IgG-HRP (7074 S) and anti-antibody-mouse IgG-HRP (7076). Blots were developed using Clarity™ Western ECL Substrate (Bio-Rad Laboratories, 1705061) and Chemidoc (Bio-Rad Laboratories) instrument, and band intensity was analyzed by ChemiDoc software (Bio-Rad Laboratories).

### Immunocytochemistry

We used vimentin (cat # ab92547) and followed our previously described immunocytochemistry protocol [59].

### Cell viability measurement by ATP assay

The luminescence levels of Adeno-Tri-Phosphate (ATP) was measured in primary cardiomyocytes and cardiac fibroblasts using CellTiter-Glo Luminescence Cell viability Assay kit (Promega, Cat # G7571) following our previously described protocol [59].

### Cell death measurement by LDH assay

Absorbance-based LDH assay was used following our previously described protocol [59].

### Treatment strategies for induction of necroptosis and inhibition

To induce necroptosis, we treated cells with TS: TNFα (T, 30 ng/ml) + Smac mimetic (S, 10 µM), and TSZ: TNFα (T, 30 ng/ml) + Smac mimetic (S, 5 µM) + Z-VAD-FMK (Z, 10 mM). To inhibit necroptosis, we pretreated cells one hour before necroptosis induction with RIPK3 inhibitor GSK872 (5 µM) and RIPK1 inhibitor Nec1 (10 µM).

### Caspase-3 activity assay

We used Caspase-Glo 3/7 Assay Promega kit and followed the kit protocol for measuring caspase-3 activity (cat #G8091).

### Caspase-8 activity assay

We used Caspase-Glo 8 Assay Promega kit and followed the kit protocol for measuring caspase-8 activity (cat # 8201).

### Enzyme-linked immuno-sorbent assay (ELISA)

We used mouse IL-1β ELISA kit (Cat # ab100705) and performws ELISA on a 100 µl of cell culture supernatant following the kit protocol. To determine the concentration of IL-1β, we measured absorbance at 450 nm using a spectrophotometer.

### MitoSOX staining

We used MitoSOX Red from Invitrogen (Cat # M36008) and followed the kit protocol to measure MitoSOX levels in the treated cells. Hoechst 33342 stain was used to counterstain the nucleus.

### Cell ROS staining

We used CellROX Green from Invitrogen (Cat # C10444) and followed the kit protocol to measure cellular ROS levels. Hoechst 33342 stain solution was used for staining the nucleus.

### Statistical analysis

All the experimental values are expressed as mean ± SEM. To compare the two groups, we used student’s *t*- test. For more than two groups, we used one-way analysis of variance (ANOVA) followed by Tukey’s multiple comparison test. We used Graph Pad Prism 9 software for statistical analyses. A *P* value <0.05 was considered statistically significant.

## Supplementary information

Supplementary Materials and Methods

Supplementary Figures

Original Data File

## Data Availability

All the experimental data are safely stored in the one drive and Box drive of the University of Nebraska Medical Center. Materials described in the manuscript, including all relevant raw data, will be freely available to any researcher wishing to use them for con-commercial purposes, without breaching participants confidentiality.
