# Supplementary material for: Differential effects of CMV infection on the viability of cardiac cells
Source: Cell Death Discov. 2023 Apr 3;9:111. doi: 10.1038/s41420-023-01408-y (PMC10070260; doi:10.1038/s41420-023-01408-y)
Supplement: Supplementary file 1 — Supplementary Materials and Methods [file 41420_2023_1408_MOESM1_ESM.docx]

**Supplementary Methods and Supplementary Figure Legends**

**Differential effects of CMV infection on the viability of cardiac cells**

Santosh K. Yadav^1^, Flobater I. Gawargi^1^, Mohammad H. Hasan^2^, Ritesh Tandon^2,3^, Jason W. Upton^4^, Paras K. Mishra^1*^

^1^Department of Cellular and Integrative Physiology, University of Nebraska Medical Center, Omaha, Nebraska, USA

^2^Department of Cell and Molecular Biology, University of Mississippi Medical Center, Jackson, Mississippi, USA

^3^Department of Medicine, University of Mississippi Medical Center, Jackson, Mississippi, USA

^4^Department of Biological Sciences, Auburn University, Alabama, USA

**Running title:** CMV in cardiac cell viability

*** Corresponding author:**

Paras Kumar Mishra, PhD, FAHA, FCVS

Department of Cellular and Integrative Physiology

University of Nebraska Medical Center

Omaha, NE-68198, USA

Phone: 402-559-8524

Fax: 402-559-4438

Email: [paraskumar.mishra@unmc.edu](mailto:paraskumar.mishra@unmc.edu)

**Isolation and culture of neonatal cardiomyocytes**

For Isolation of neonatal cardiomyocytes, 1–2-day old pups from C57BL/6J mouse were decapitated and hearts were dissected out. Primary cardiomyocytes were isolated using Pierce Primary Cardiomyocyte Isolation Kit (Cat # 88281) and following the kit protocol. Briefly, cardiomyocyte isolation enzyme 1 was reconstituted in 2.4 ml Ca^++^/Mg^++^ free HBSS solution. Freshly dissected hearts were washed one time in the ice-cold HBSS and then transferred into a 1.5 ml microfuge tube containing 0.5 ml of ice-cold HBSS. Hearts were minced into 1-3 mm^3^ pieces and washed two times in ice-cold HBSS. Each tube containing the minced heart tissue was mixed with 200 µl of cardiomyocyte isolation enzyme 1 and 10 µl of cardiomyocyte isolation enzyme 2. The tubes were gently shacked to mix the enzyme solutions with the minced tissue and then incubated at 37^0^C for 30-35 min. After the incubation, enzyme solutions were gently removed, and the tissue was washed two times with 500 µl of ice-cold HBSS. After washing, 500 µl DMEM primary cell isolation medium was added to each tube and the washed tissue was further fragmented into small pieces by pipetting up and down 25-30 times using a 1 ml sterile pipette tip. This results into formation of a single-cell suspension. In each tube, 1 ml complete medium was added to make the final volume to 1.5 ml. Cells of all the tubes were pooled into one tube to measure the yield of the isolated cells. The cell viability was measured by trypan blue staining and cell counting. After assessing yield and viability, cells were cultured in a 6-well culture plate. The next day, culture medium was replaced with a complete DMEM medium containing 1X cardiomyocyte growth supplement. After two days of culture, experiments were performed.

**Isolation and culture of neonatal cardiac fibroblast**

We used 4–5-week-old C57BL/6J mouse hearts for isolation of cardiac fibroblasts. For extracting the heart, we used same method that was used for isolating primary cardiomyocytes. Hearts were washed one time with ice-cold HBSS and transferred into a small new petri dish containing ice-cold HBSS. Hearts were minced in 1-3 mm^3^ pieces and washed in ice-cold HBSS. Minced tissue was mixed with 3-4 ml (depending upon tissue size) of 0.1% collagenase II in a 50 ml falcon tube and incubated at 37^0^C for 45-60 min in a shaker incubator. Tubes were shaken vigorously every 15-20 min to enhance lysis. At the end of incubation, an equal volume of complete DMEM medium (high glucose DMEM+10% FBS+ 1% Pen/Strep) was added to the tubes to stop the enzymatic tissue lysis. Lysed tissue was washed two times in ice-cold HBSS. After washing, the tissue pellet was resuspended in complete DMEM medium. Using a 1 ml pipette, we had pipetting up and down culture medium several times to make a single-cell suspension. Then, we assessed the yield of the cells by trypan blue staining and cell counting and plated them in a culture plate with complete DMEM medium containing 5 ng/ml FGF growth factor. After 24 h, medium was replaced with a fresh medium and culture until they attained desired confluency for experiments.

**Protein isolation and Western blotting**

# To extract protein from the cell pellet, we incubated cells with radio-immuno-precipitation assay (RIPA) lysis buffer (BP-115D, Boston BioProducts) for 30 minutes on ice. The cell lysate was centrifuged at 10000 rpm at 4^o^C for 20 minutes and supernatant was collected for protein estimation using the BCA method (Pierce BCA Protein Assay Kit, 23227, Thermo Fisher). We loaded 20-25 µg of protein for SDS PAGE. Proteins on the gel were transferred on to a nitrocellulose membrane. After protein transfer, the membrane was blocked with 5% nonfat milk for 1 h and incubated with primary antibody overnight at 4^o^C on a rocker. The primary antibodies used were pRIPK3 (Cat # 91702), RIPK3 (Cat # ab56164), pRIPK1 (Cat # 65746), ZBP1 (Cat # NBP1-76854), IE1 (anti-m123/IE1- clone IE1.01, Cat # R-MCMV-12), E1 (anti- M112-113/E1- clone- chroma-103, Cat # HR-MCMV-07), gB (anti- M55/gB- clone-M55.01, Cat # HR-MCMV-05), and β-actin (ab56164). The membrane was washed with TBST three times at 5 min intervals. They were incubated with secondary antibodies for 1 h at RT. The dilution of the secondary antibody was double of the primary antibody dilution. The secondary antibodies used were anti-rabbit IgG-HRP (7074S) and anti-mouse IgG-HRP (7076). Blots were developed using Clarity™ Western ECL Substrate (Bio-Rad Laboratories, 1705061) and Chemidoc (Bio-Rad Laboratories) instrument and band intensity was analyzed by ChemiDoc software (Bio-Rad Laboratories).

**Cell viability measurement by ATP assay**

Primary neonatal cells, 3X10^4^ cells were seeded in a 96-well plate and after two days of culture, cells were infected with the virus. For cardiac fibroblast, 5X10^3^ cardiac fibroblast cells isolated from the C57BL/6J mouse heart were seeded in a 96-well plate. After 24 h, cells were infected with WT and different mutant MCMV viruses at MOI = 5 for 24 hours. At the end of infection, cell viability was determined by measuring luminescence (ATP level) using CellTiter-Glo Luminescence Cell viability Assay kit (Promega, Cat # G7571) following the kit protocol. Culture medium (without cells) was used as a background control for the luminescence assay. The values were presented as a relative luminescence intensity (RLU).

**Cell death measurement by LDH Assay**

Lactate dehydrogenase (LDH) assay was performed to measure cell cytotoxicity. Cell culture medium (50 µl) was added to a 96-well flat-bottom plate. After that 50 µl LDH reaction mixture was added to each sample well. The medium and LDH were mixed by a gentle tapping or pipetting without generating bubbles. The plate was incubated at the room temperature for 30 min in dark. At the end of incubation, the reaction was stopped by addition of 50 µl of the stop solution to each well and mixing them well by gentle tapping or pipetting without generating bubbles. This solution was used for absorbance at 490 nm and 690 nm using a spectrophotometer. LDH activity was determined by subtracting the 680 nm absorbance value (background signal) from the 490 nm absorbance value. The values were presented as background-subtracted absorbance values at 490 nm relative to the value of untreated control.

**Treatment strategies for the induction and inhibition of necroptosis**

For induction of necroptosis, 5X10^3^ HL1 cells were seeded in a 96-well plate in complete medium. After 24 h of incubation in a 5% CO_2_ incubator at 37^ο^C, cells were infected with MCMV and treated with TS, TNFα (T, 30 ng/ml) + Smac mimetic (S, 10 µM) as well as TSZ, TNFα (T, 30 ng/ml) + Smac mimetic (S, 5 µM) + Z-VAD-FMK (Z, 10 mM). The DMSO-treated cells were used as a control (uninfected/MOCK) cell. For the inhibition assay, RIPK3 inhibitor GSK872 (5 µM) and RIPK1 inhibitor Nec1 (10 µM) were pretreated 1 h before the treatment of TS, TSZ, and DMSO (MOCK). The treatment duration was 24 h. At the end of treatment, cell viability was determined using the CellTiter-Glo Luminescence Cell viability Assay kit. The culture medium (without cells) was used as background luminescence. The values were presented as a relative luminescence intensity (RLU).

**Caspase-3/7 activity assay**

Primary neonatal cells (3X10^4^) were seeded in a 96-well plate culture medium. After 48 h of culture, cells were infected with MCMV and mutant MCMV at MOI = 5 for 24 h. After 24 h, caspase-3 activity was measured using the caspase3/7 Glo kit following the kit protocol.

**Caspase-8 activity assay**

The same protocol that was used for caspase-3/7 treatment was followed. At the end of treatment, caspase-8 activity was measured using Caspase-Glo 8 assay kit (Promega) following the kit protocol.

**Enzyme-linked immunosorbent assay (ELISA)**

We determined the levels of Interleukin-1 beta (IL-1β) and TNFα in culture medium using IL-1β (Cat # ab 100705) and mouse TNFα (cat # DY410-05) ELISA kit. We added 100 µl of culture medium from the treated/infected cells to the assay wells provided in the kit. Wells were covered and incubated for 2.5 h at room temperature with a gentle shaking. After that, medium was discarded, and the well was washed 4 times with 1X wash solution. After the last wash, the remaining wash buffer was removed by aspirating and then blotted against the clean towel paper. After blotting, 100 µl of 1X biotinylated IL-1β or TNFα detection antibody was added to each well as per experimental design. Wells were incubated for 1 h at room temperature with a gentle shaking and then the antibody was discarded, and the wells were washed 4 times with 1X washing solution. After that 100 µl 1X HRP-streptavidin solution was added to each well and incubated for 45 minutes at room temperature with a gentle shaking. Then, the wells were washed 4 times with 1X washing solution. After washing, 100 µl TBM one-step substrate reagent was added to each well. Wells were incubated for 30 minutes at room temperature with a gentle shaking. After incubation, 50 µl stop solution was added to each well. Absorbance was measured immediately at 450 nm in a spectrophotometer and the concentration of cytokines was calculated.

**MitoSOX staining**

Mitochondrial superoxide (MitoSox) level was measured by MitoSOX Red kit from Invitrogen (cat # M36008). After isolating primary neonatal cardiomyocytes, a total number of 0.3 X10^6^ cells were seeded in a 24-well plate. After 48 h of culture, cells were infected with WT MCMV and mutants of MCMV at MOI = 5 for 24 h. At the end of incubation, cells were stained with MitoSOX Red reagents as per the kit protocol. Hoechst 33342 stain was used to counterstain the nucleus. After staining, cells were analyzed under an EVOS microscope for imaging at 510/580 Ex/Em.

**Cell ROS staining**

Cellular oxidative stress was measured by CellROX Green kit from Invitrogen (cat # C10444). After isolating primary neonatal cardiomyocytes, a total number of 0.3 X10^6^ cells were seeded in a 24-well plate. After 48 h of culture, cells were infected with WT and mutant MCMVs at MOI = 5 for 24 h. At the end of incubation, cells were stained with 5 µM CellROX Green reagent for 30 minutes as per kit protocol. Hoechst 33342 stain solution was used for staining the nucleus. After staining, cells were analyzed under an EVOS microscope or imaging at 485/520 Ex/Em.

.

**Supplementary Figure Legends**

**Figure S1. Standardization of dose and time for MCMV infection in HL1 cells. A-B.** Phase contrast imaging showing the morphology of HL1 cells after infection with different dose and time of MCMV. **C.** The titer of MCMV after infecting HL1 cells with different MOI. **D.** Cell viability measurement through ATP assay in uninfected (MOCK) and MCMV infected HL1 cells at three time points. Student’s t-test was performed. Values are mean ± SE. Each point represents one sample. n= 3-8. *; *P*<0.05; ****; *P*<0.0001.

**Figure S2. MCMV infection promotes cell survival and inhibits cell death which is independent of its necroptotic activity in HL1 cells. A.** Phase contrast images of HL1 cells infected with MOCK, WT CMV and M45*mut* RHIM MCMV for 24 h. **B.** Measurement of cell viability by ATP assay. **C.** Measurement of cytotoxicity by lactate dehydrogenase (LDH) assay using cell culture medium. One-way ANOVA followed by Tukey’s multiple comparison test was performed. Values are mean ± SE. Each point represents one sample. n= 6-8. **; *P*<0.01; ****; *P*<0.0001.

**Figure S3. Validating MCMV infection using uninfected (MOCK) and WT and M45*mut*RHIM MCMV infected HL1 cells. A.** Immunoblotting and quantification of Immediate Early-1 (IE-1) viral protein showing successful infection of MCMV. **B.** Immunoblotting and quantification of Early-1 (E-1) viral protein. **C.** Immunoblotting and quantification of envelop glycoprotein (gB) showing replication of MCMV. One-way ANOVA followed by Tukey’s multiple comparison test was performed. Values are mean ± SE. Each point represents one sample. n= 6. **; *P*<0.01; ***; *P*<0.001; ****; *P*<0.0001.

**Figure S4. MCMV (both WT and M45*mut*RHIM) infection decreases cytosolic and mitochondrial ROS in HL1 cells. A.** Immunofluorescence and quantification of cellular ROS in uninfected (MOCK), WT and M45*mut*RHIM MCMV infected HL1 cells. **A.** Immunofluorescence and quantification of mitochondrial ROS in uninfected (MOCK), WT and M45*mut*RHIM MCMV infected HL1 cells. One-way ANOVA followed by Tukey’s multiple comparison test was performed. Values are mean ± SE. Each point represents one sample. n= 4-5. *; *P*<0.05; **; *P*<0.01; ****; *P*<0.0001.

**Figure S5. MCMV (both WT and M45*mut*RHIM) infection promotes mitochondrial biogenesis and mitochondrial mass in HL1 cells. A.** Immunoblotting and quantification of mitochondrial biogenesis marker - peroxisome proliferator-activated receptor coactivator 1-alpha (PGC1α) - in the uninfected (MOCK) and MCMV infected HL1 cells. **B.** Immunoblotting and quantification of mitochondrial protein - transcription factor A, mitochondrial (TFAM) - in the uninfected (MOCK) and MCMV infected HL1 cells. One-way ANOVA followed by Tukey’s multiple comparison test was performed. Values are mean ± SE. Each point represents one sample. n= 5-6. *; *P*<0.05; **; *P*<0.01; ***.

**Figure S6. CMV infection induces TNFα in cardiac fibroblast.** Phase-contrast imaging of uninfected and MCMV infected cardiac fibroblasts and quantification of tumor necrosis factor-alpha (TNFα) in the culture medium. Student’s t-test was performed. Values are mean ± SE. Each point represents one sample. n= 6-7. **; *P*<0.01.

**Figure S7. ZBP1 variant in cardiomyocytes. A.** Immunoblot and quantification of ZBP1 with molecular weight of 42 kDa in cardiomyocyte cell line HL1. One-way ANOVA followed by Tukey’s multiple comparison test was performed. Values are mean ± SE. Each point represents one sample. n= 6.

**Fig. S8. Splice variants of mouse Zbp1. A.** A snapshot from NCBI showing splice variants of mouse Zbp1. **B.** Presence and absence of RHIM domains in two splice variants of mouse Zbp1**.** RHIM motif sequences for Zbp1 was obtained from Uniprot database Q9QY24, Q9QZL0, respectively. Serial Cloner software was used to localize the motifs sequence in the mRNA and protein sequences. Bio Render was used to create the graphical diagrams.

**Figure S9. ZBP1 interacting proteins.** List of proteins that are documented to interact with ZBP1.
