# Supplementary material for: Differential effects of CMV infection on the viability of cardiac cells
Source: Cell Death Discov. 2023 Apr 3;9:111. doi: 10.1038/s41420-023-01408-y (PMC10070260; doi:10.1038/s41420-023-01408-y)
Supplement: Supplementary file 2 — Supplementary Figures [file 41420_2023_1408_MOESM2_ESM.pptx]

## Slide 1
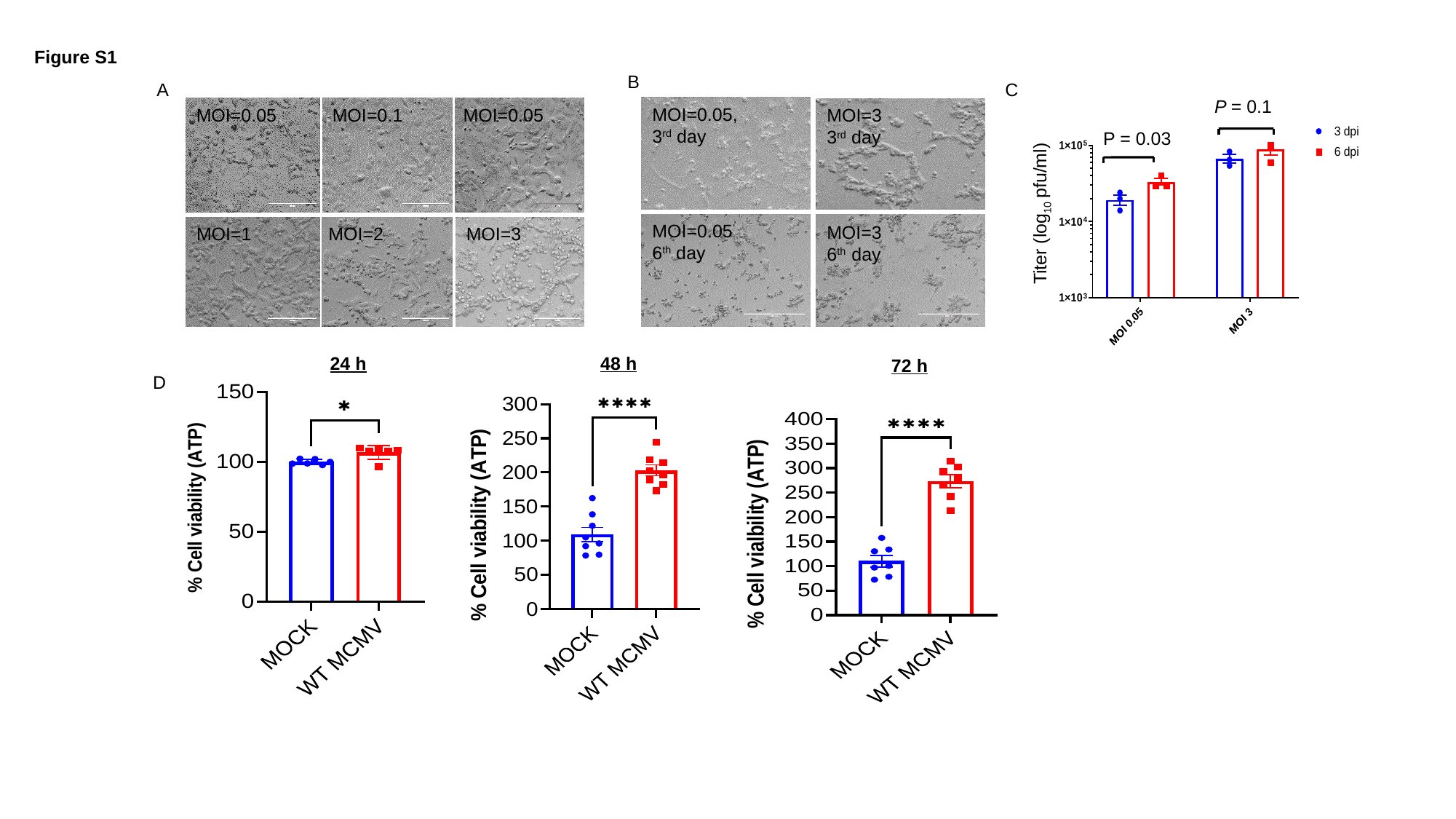

Figure S1
B
A
C
P = 0.1
P = 0.03
Titer (log10 pfu/ml)
MOI=0.05, 3rd day
MOI=3
3rd day
MOI=0.05
6th day
MOI=3
6th day
MOI=0.05
MOI=0.1
MOI=0.05
MOI=3
MOI=2
MOI=1
48 h
24 h
72 h
D

## Slide 2
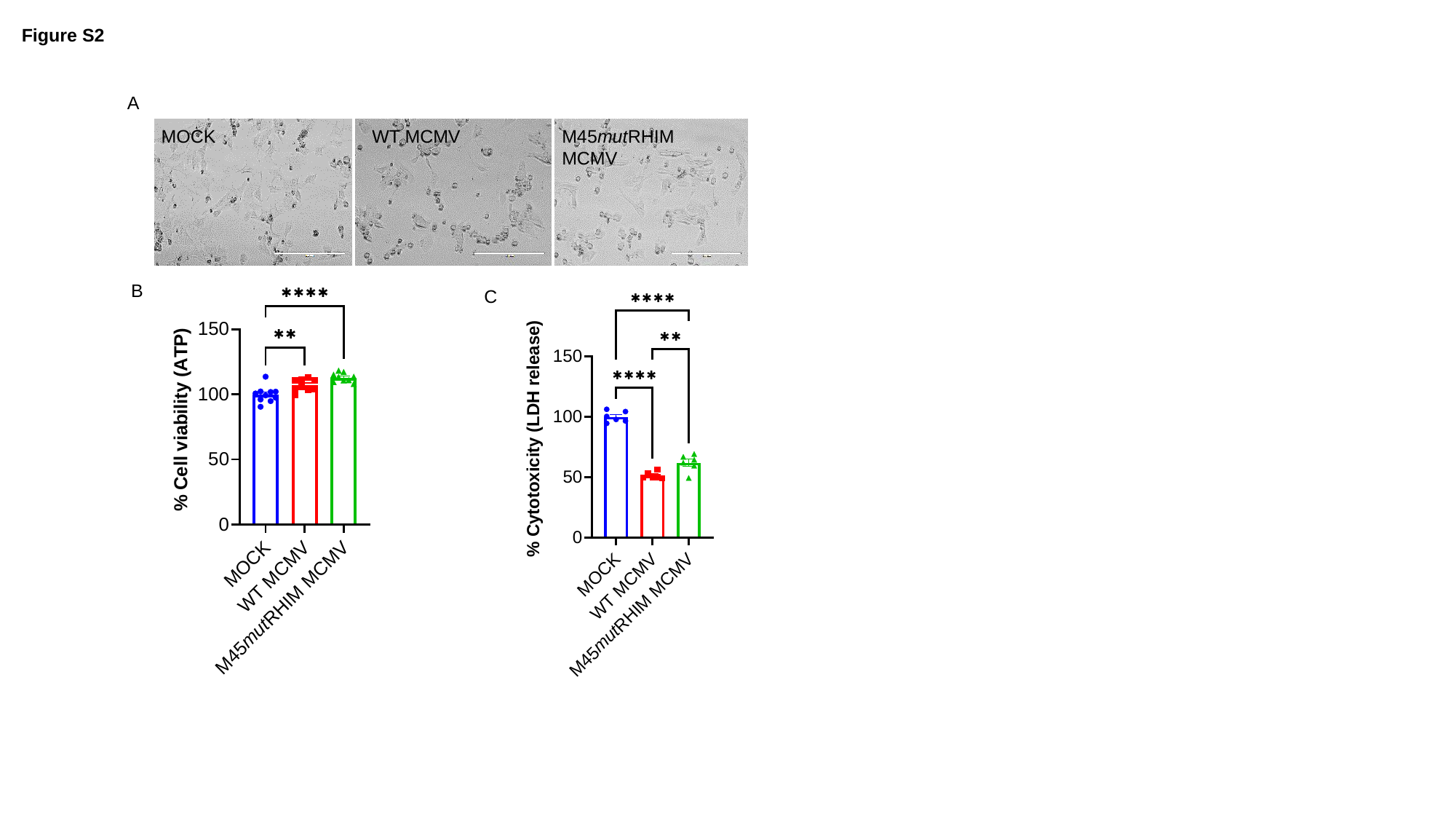

Figure S2
A
WT MCMV
MOCK
M45mutRHIM MCMV
B
C

## Slide 3
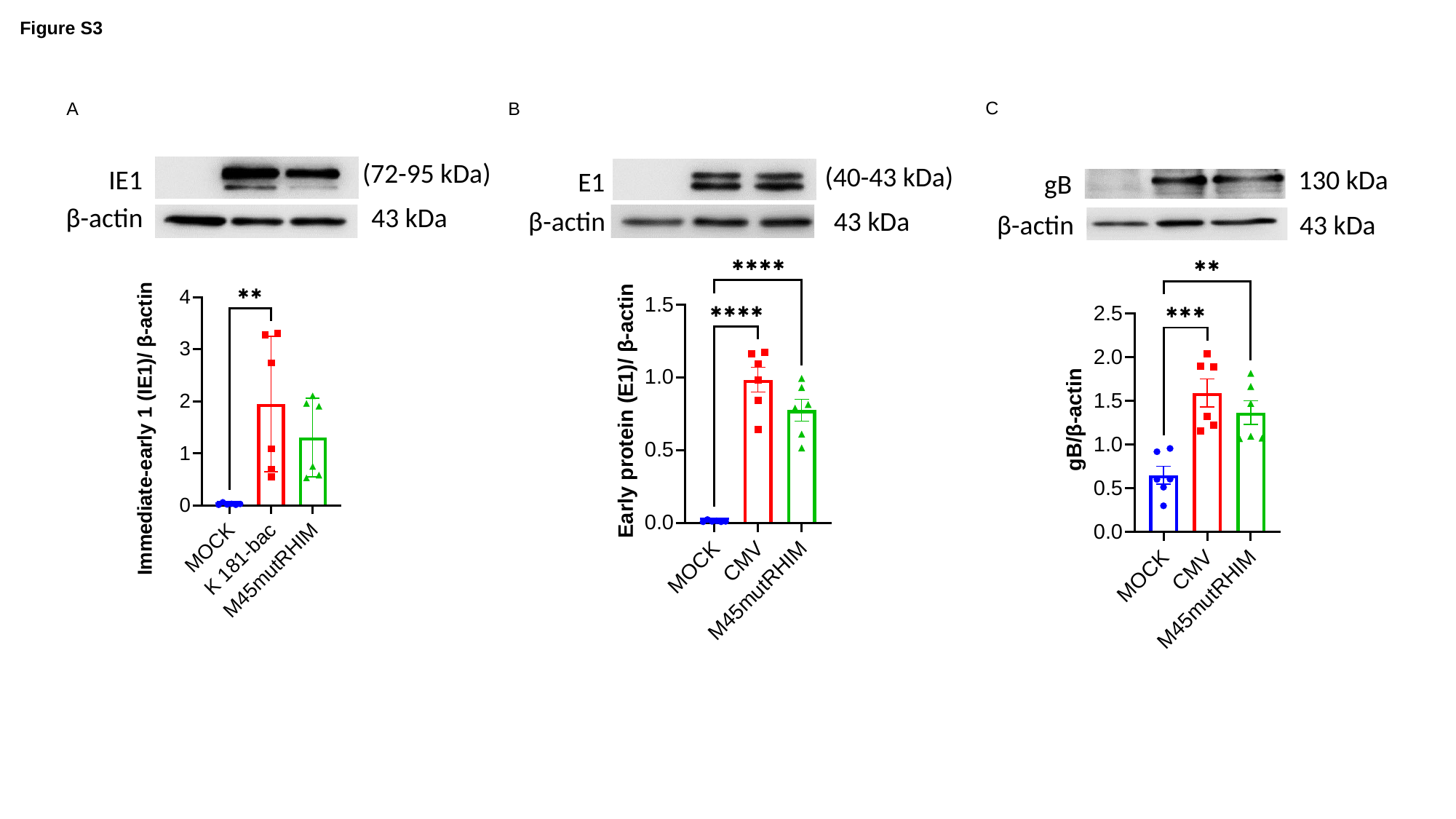

Figure S3
C
A
B
(72-95 kDa)
IE1
β-actin
43 kDa
(40-43 kDa)
E1
β-actin
43 kDa
130 kDa
gB
β-actin
43 kDa

## Slide 4
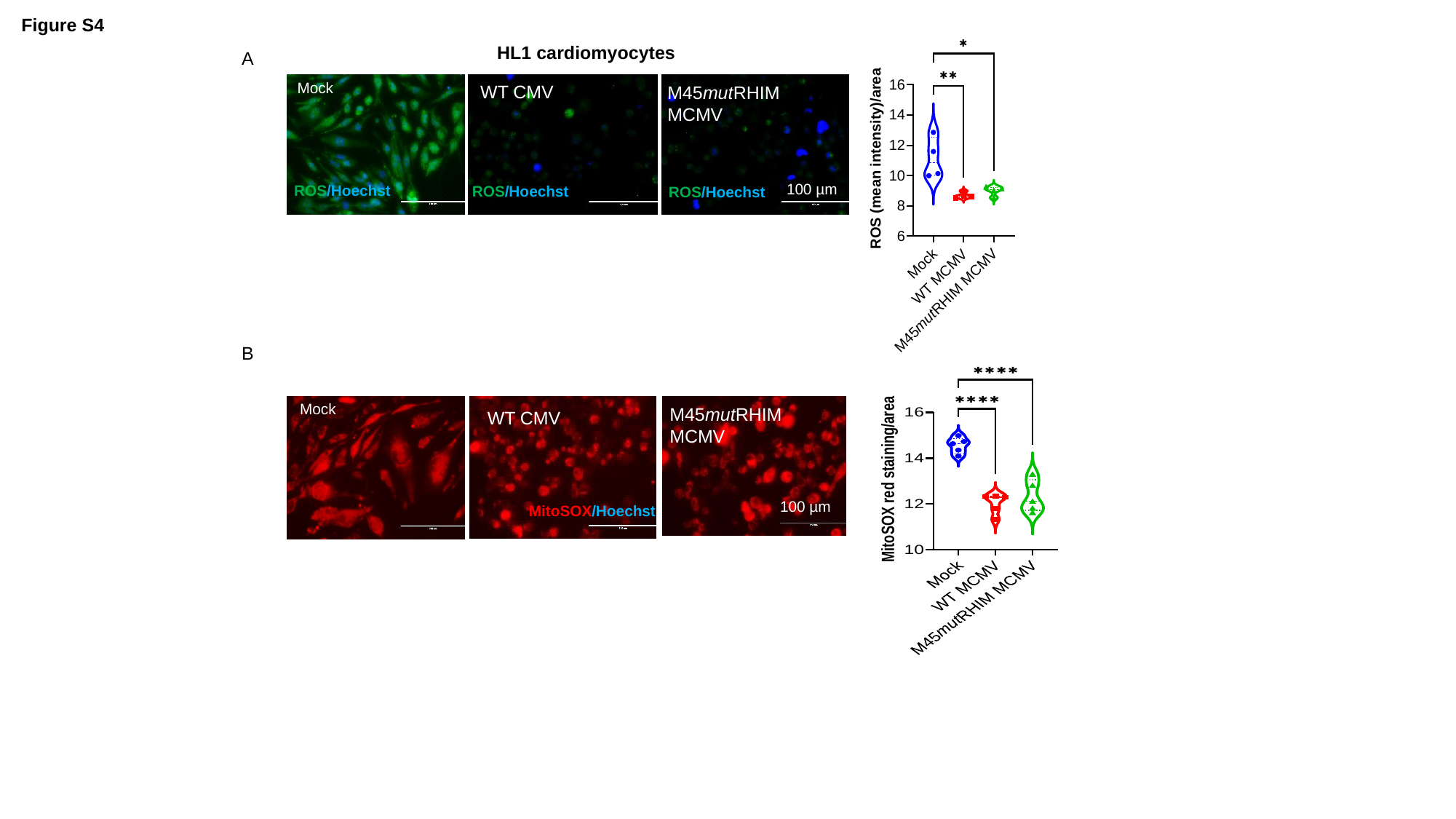

Figure S4
HL1 cardiomyocytes
A
Mock
WT CMV
M45mutRHIM MCMV
100 µm
ROS/Hoechst
ROS/Hoechst
ROS/Hoechst
B
Mock
100 µm
MitoSOX/Hoechst
M45mutRHIM MCMV
WT CMV

## Slide 5
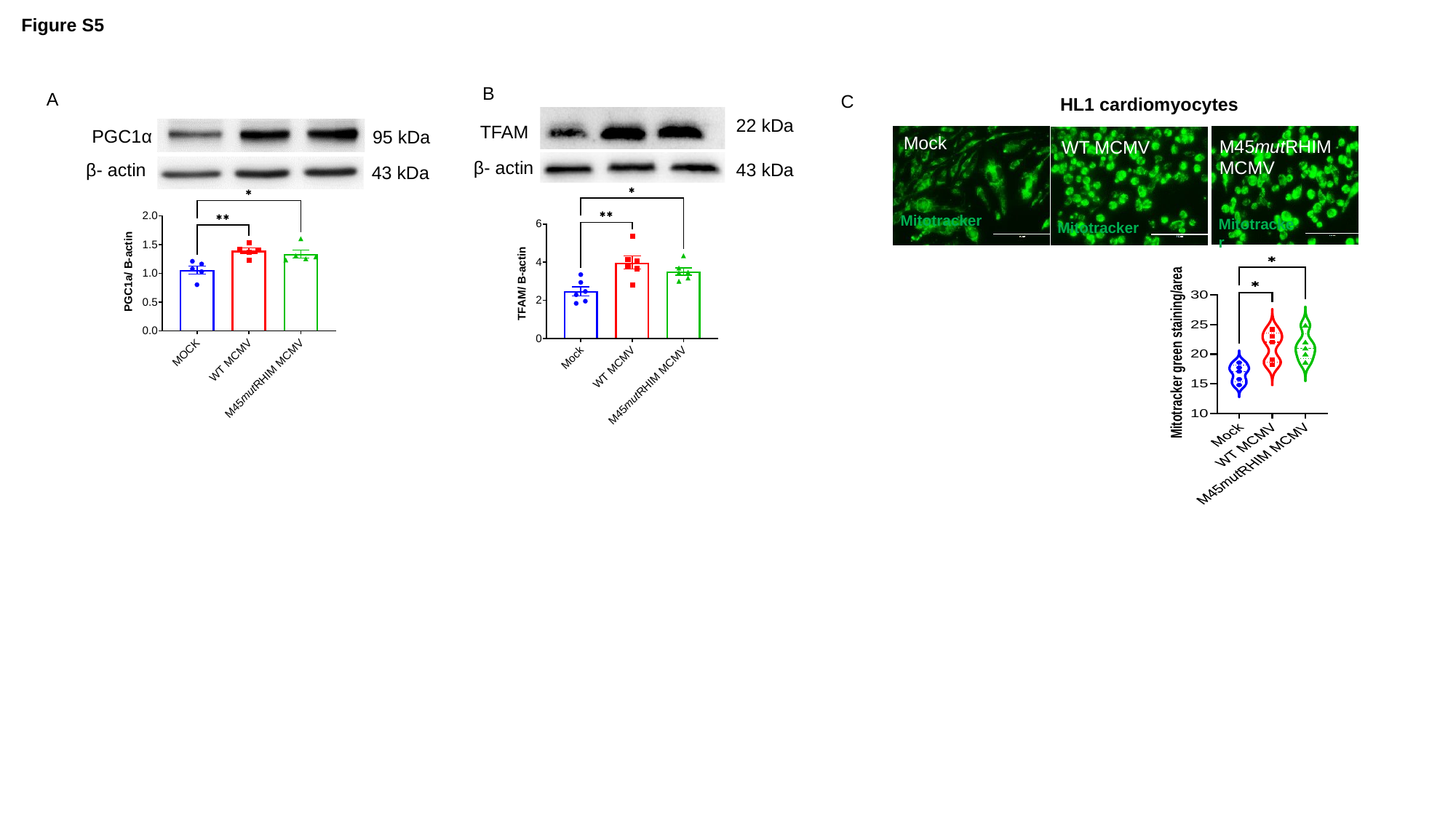

Figure S5
B
A
C
HL1 cardiomyocytes
Mock
M45mutRHIM
MCMV
WT MCMV
Mitotracker
Mitotracker
Mitotracker
22 kDa
TFAM
β- actin
43 kDa
PGC1α
95 kDa
β- actin
43 kDa

## Slide 6
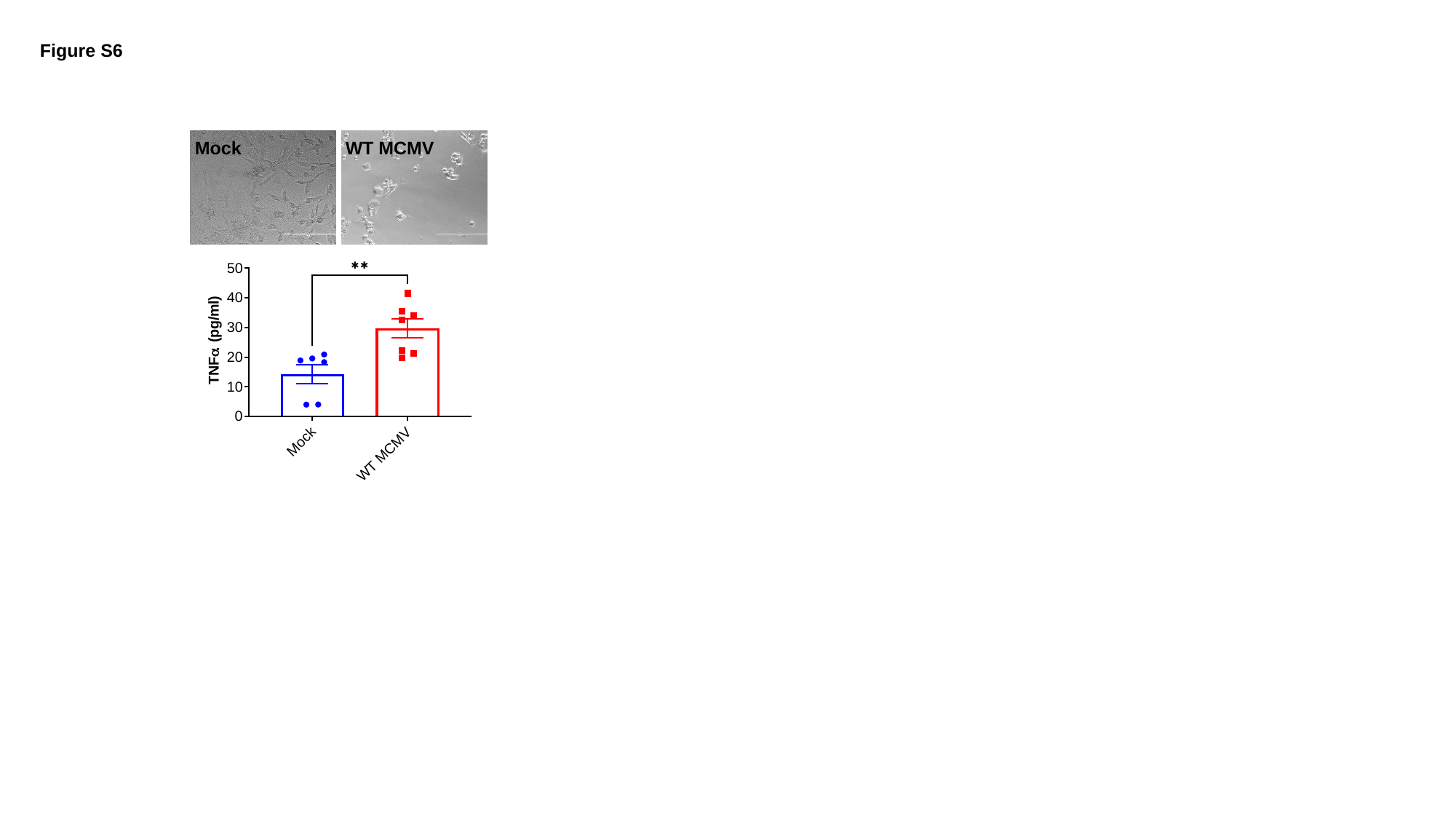

Figure S6
WT MCMV
Mock

## Slide 7
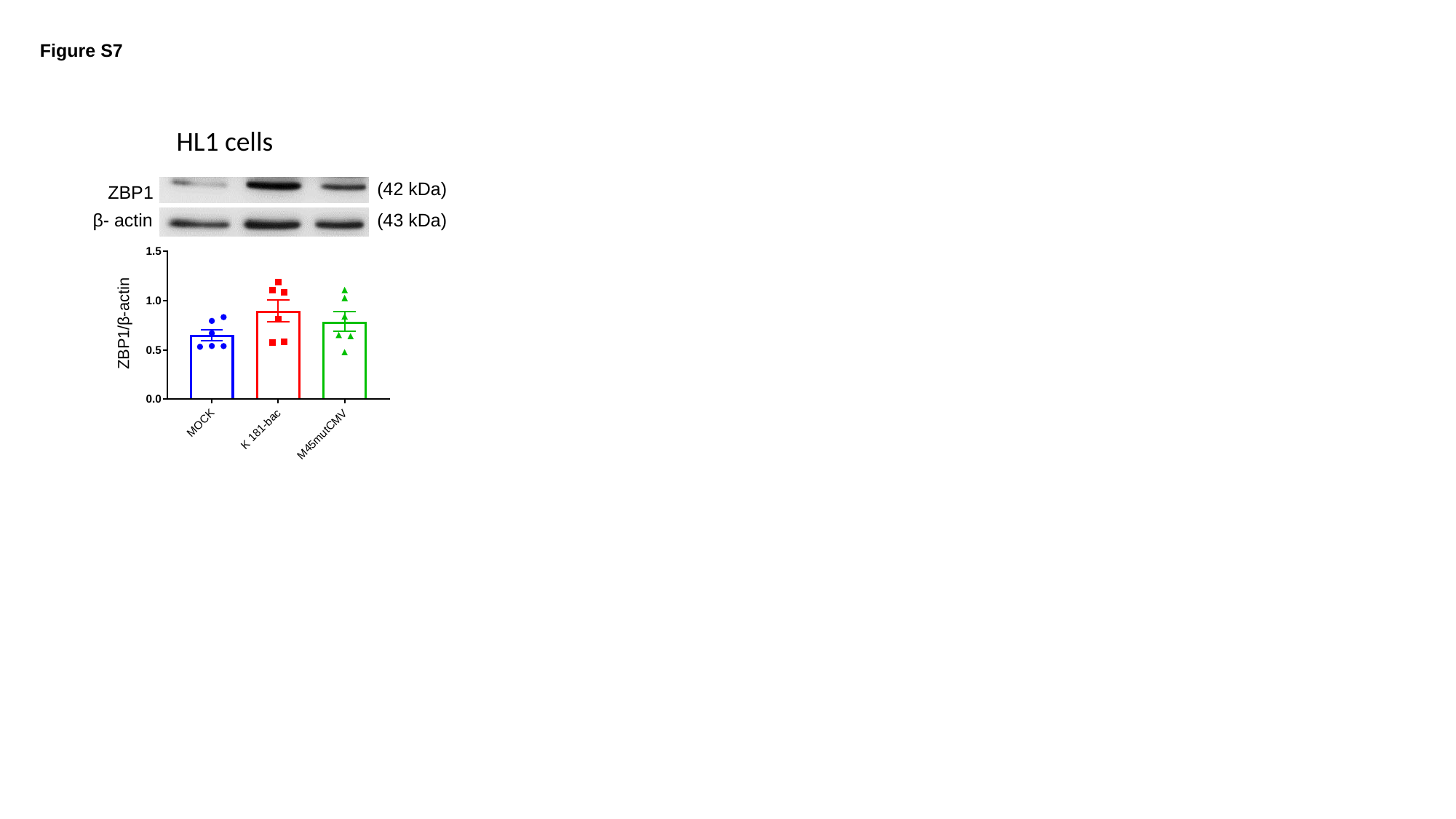

Figure S7
HL1 cells
(42 kDa)
 ZBP1
β- actin
(43 kDa)
ZBP1/β-actin

## Slide 8
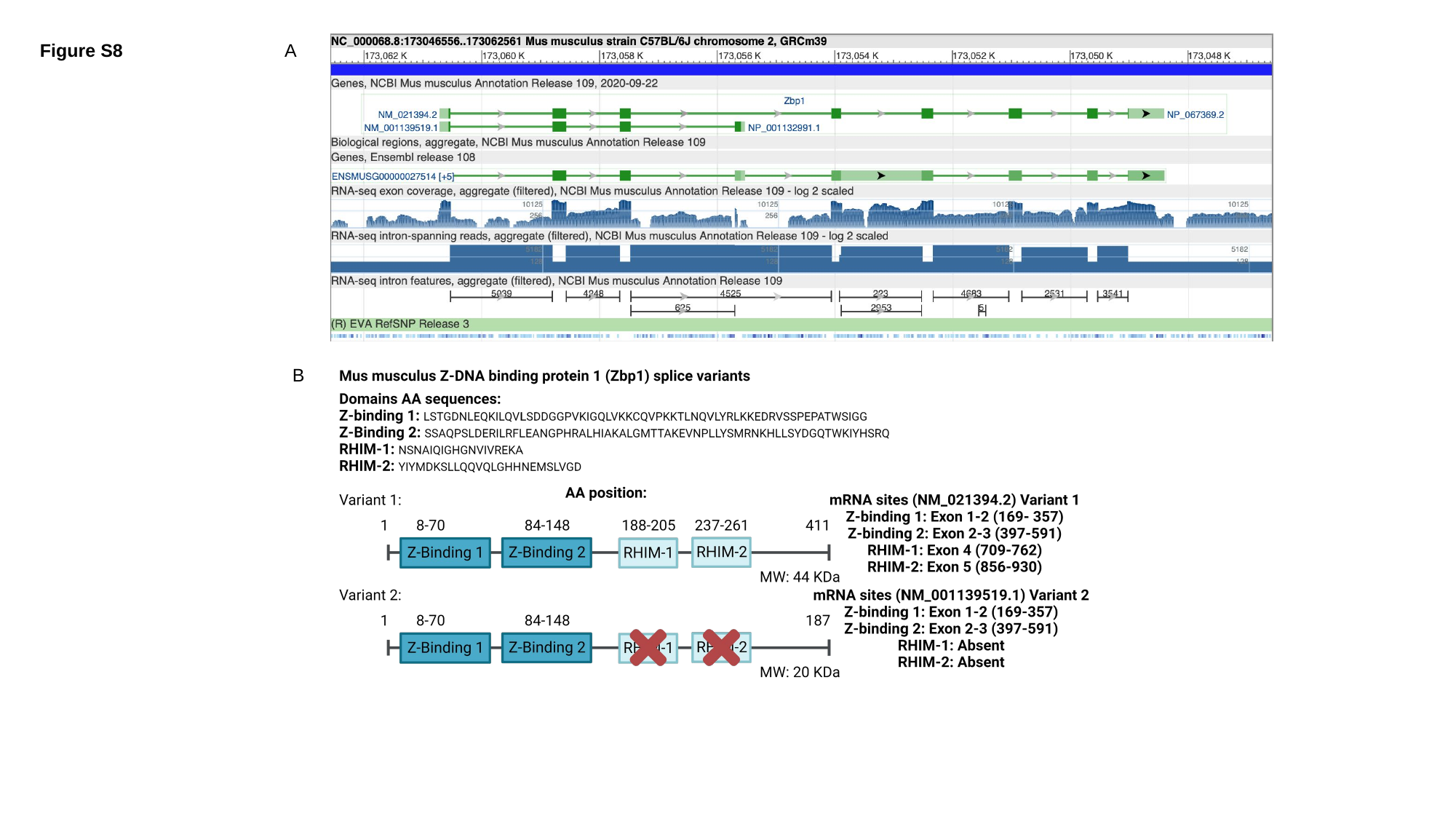

Figure S8
A
B

## Slide 9
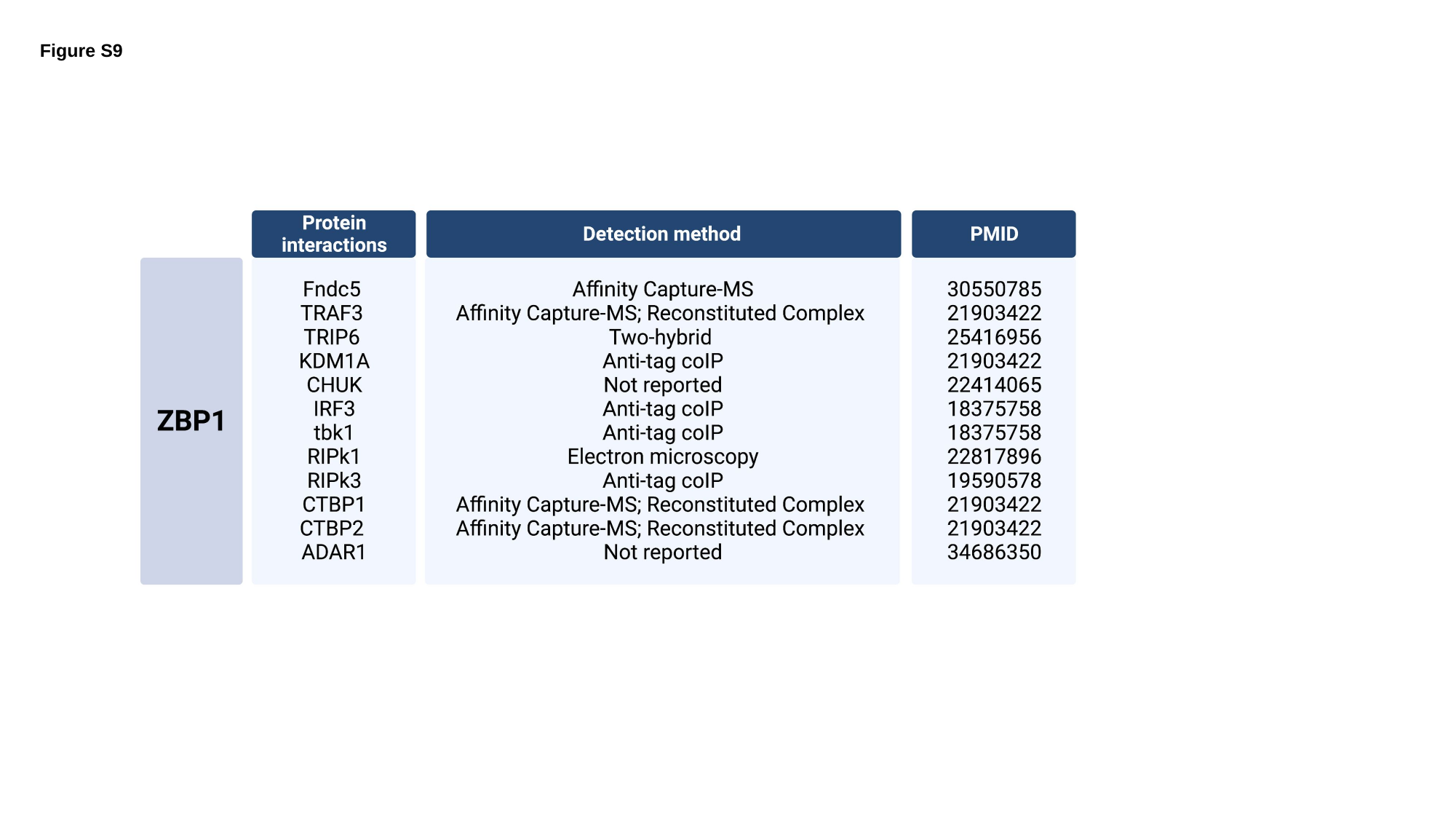

Figure S9
