# Supplementary material for: Differential effects of CMV infection on the viability of cardiac cells
Source: Cell Death Discov. 2023 Apr 3;9:111. doi: 10.1038/s41420-023-01408-y (PMC10070260; doi:10.1038/s41420-023-01408-y)
Supplement: Supplementary file 3 — Original Data File [file 41420_2023_1408_MOESM3_ESM.pptx]

## Slide 1
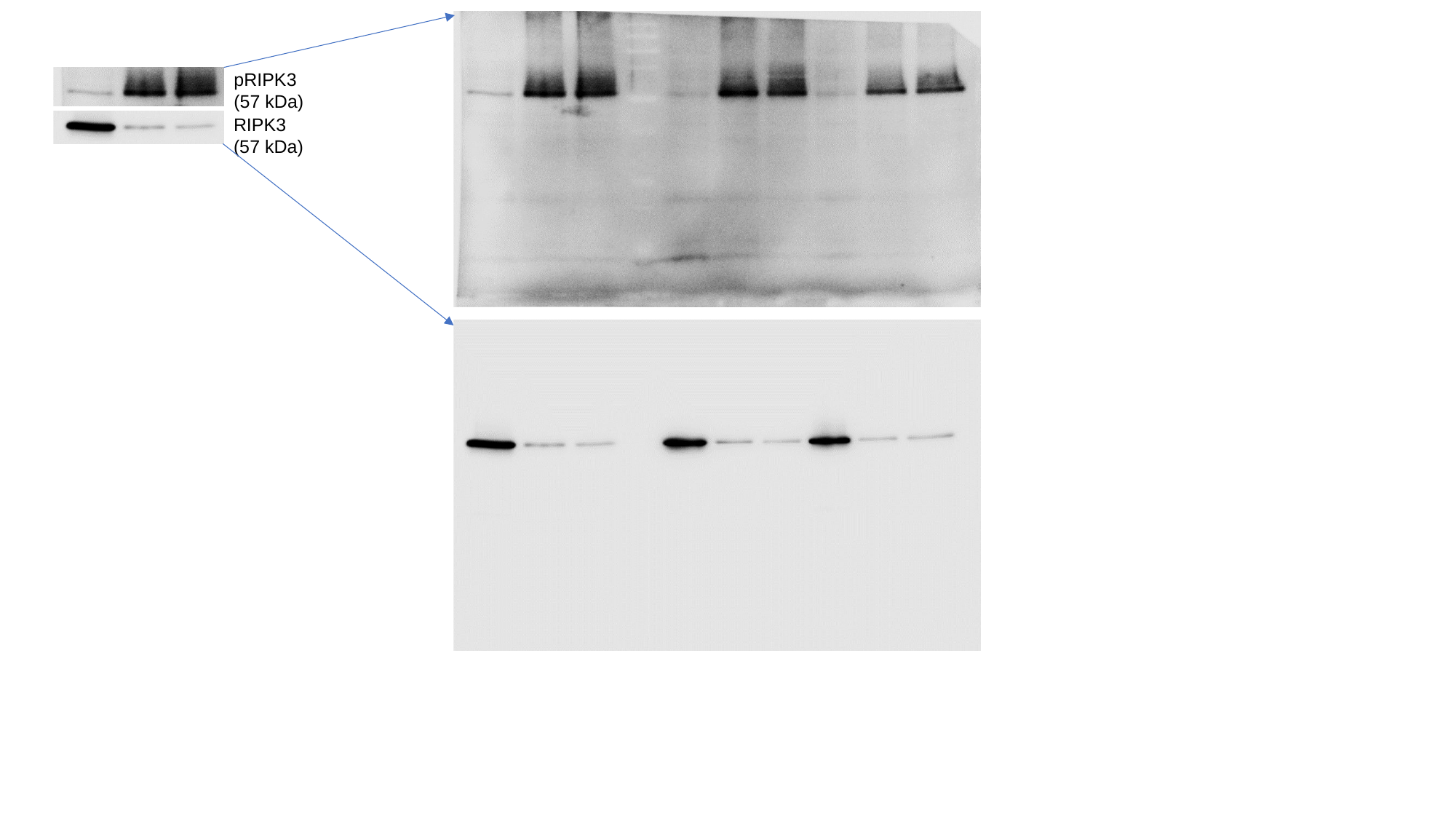

pRIPK3
(57 kDa)
RIPK3
(57 kDa)

## Slide 2
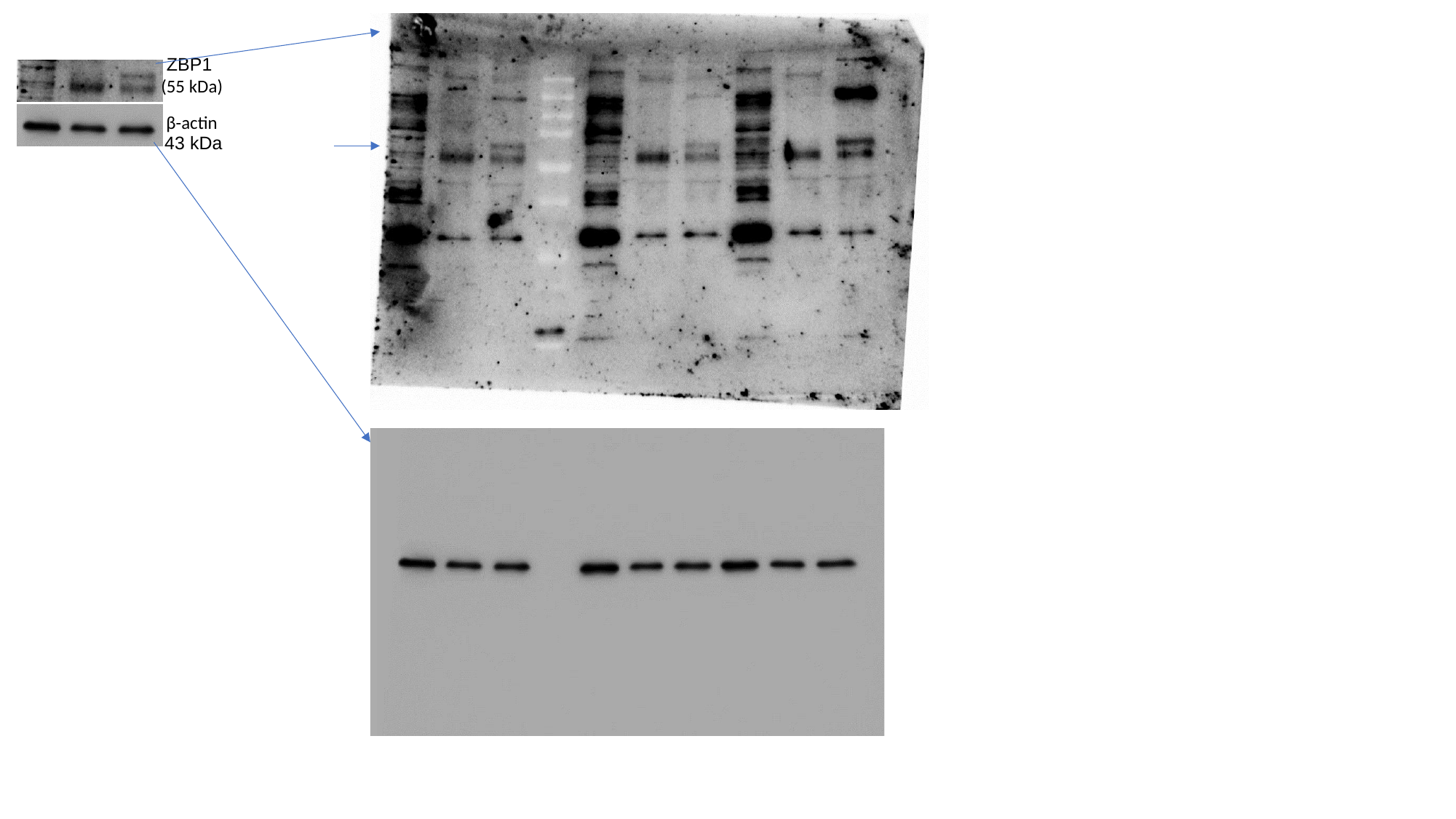

ZBP1
(55 kDa)
β-actin
43 kDa

## Slide 3
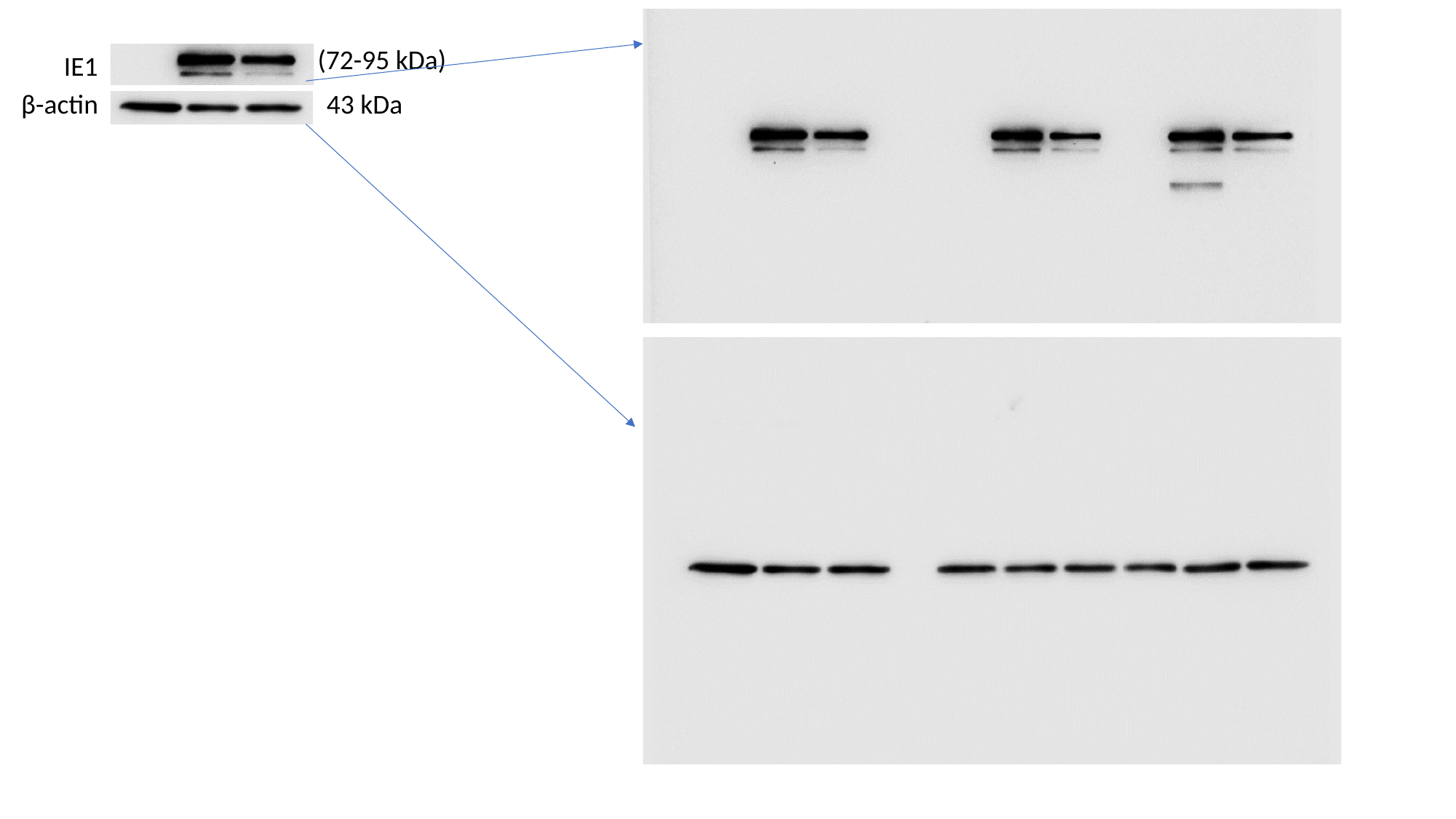

(72-95 kDa)
IE1
β-actin
43 kDa

## Slide 4
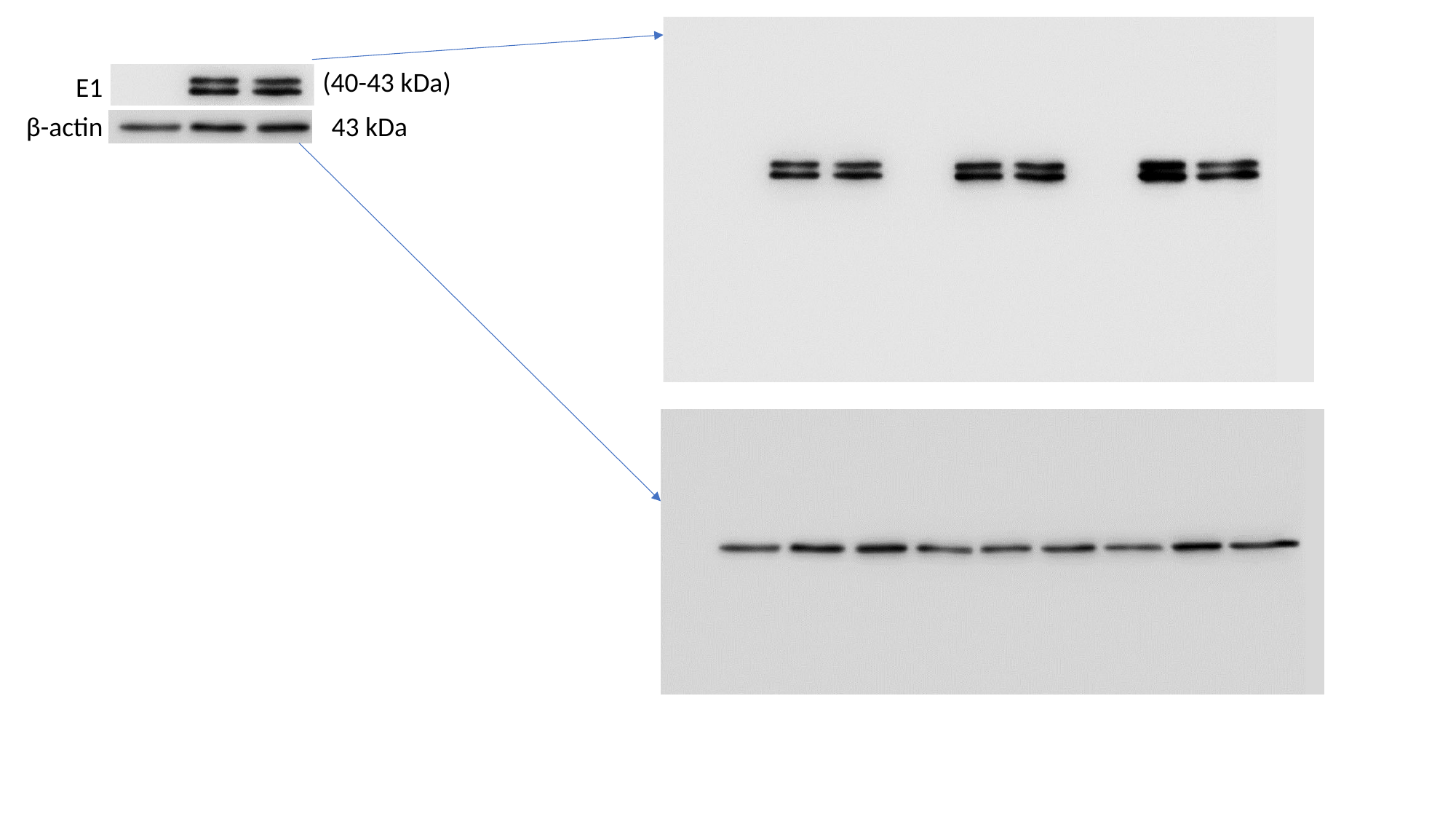

(40-43 kDa)
E1
β-actin
43 kDa

## Slide 5
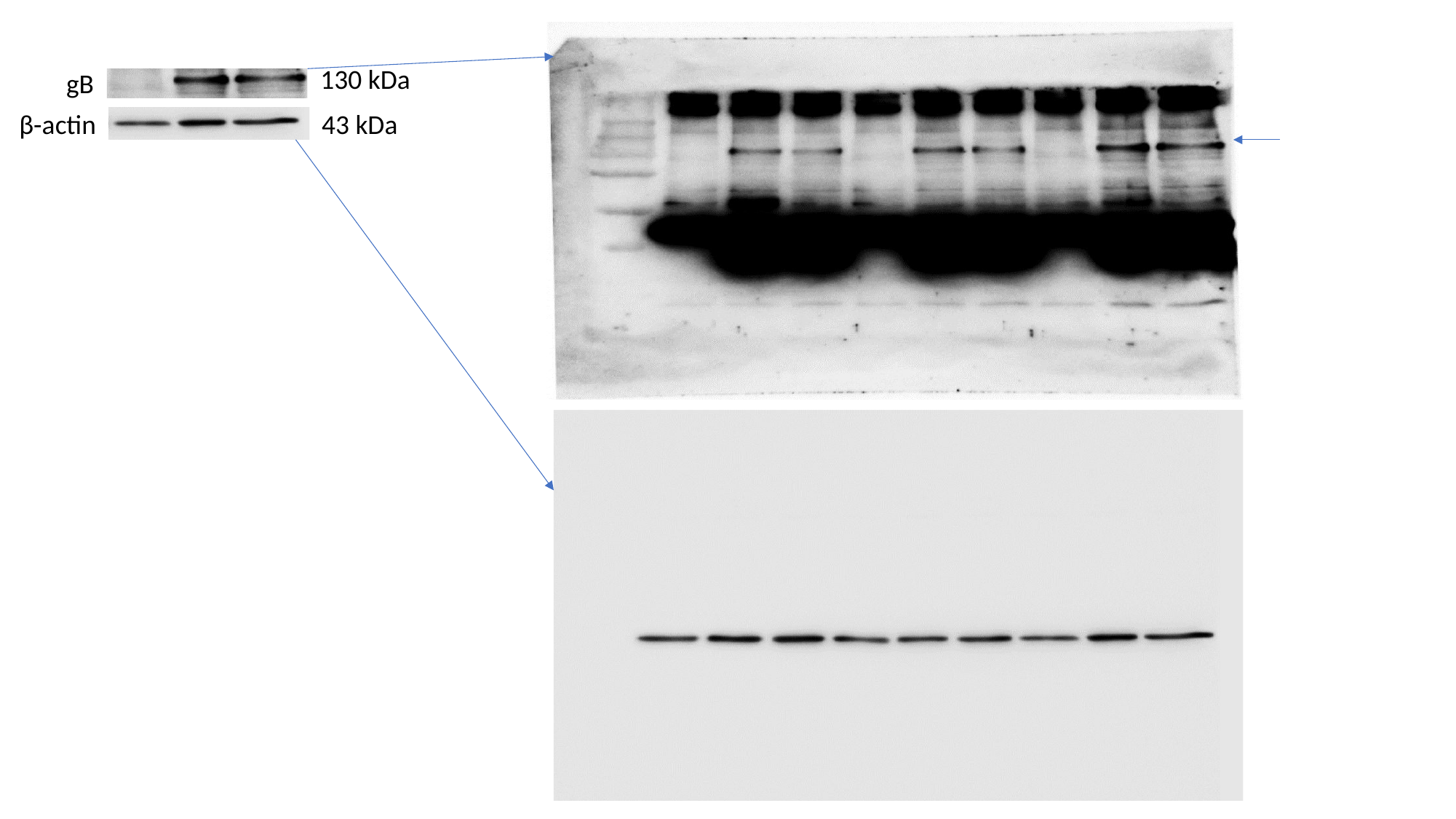

130 kDa
gB
β-actin
43 kDa

## Slide 6
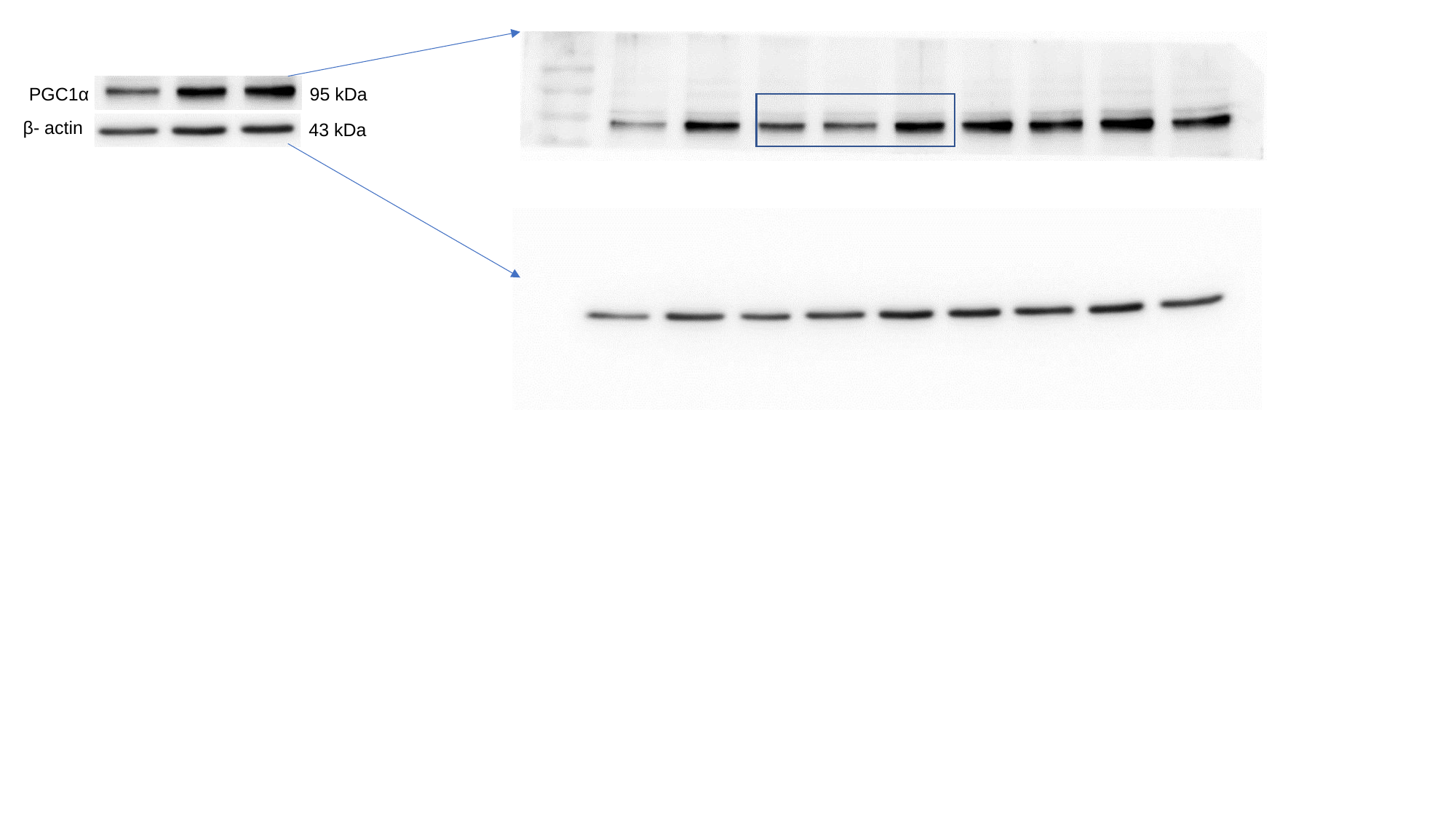

PGC1α
95 kDa
β- actin
43 kDa

## Slide 7
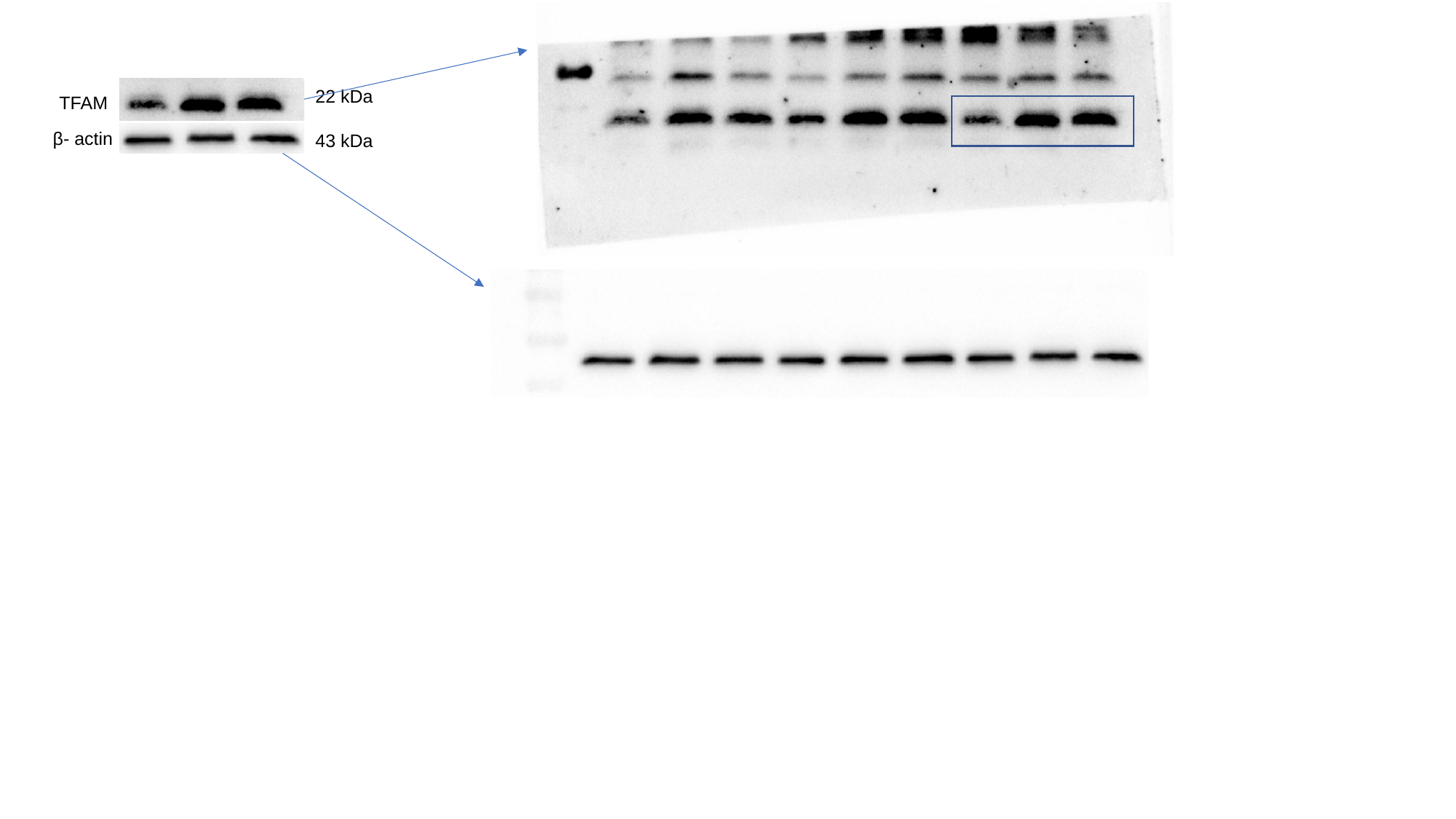

22 kDa
TFAM
β- actin
43 kDa

## Slide 8
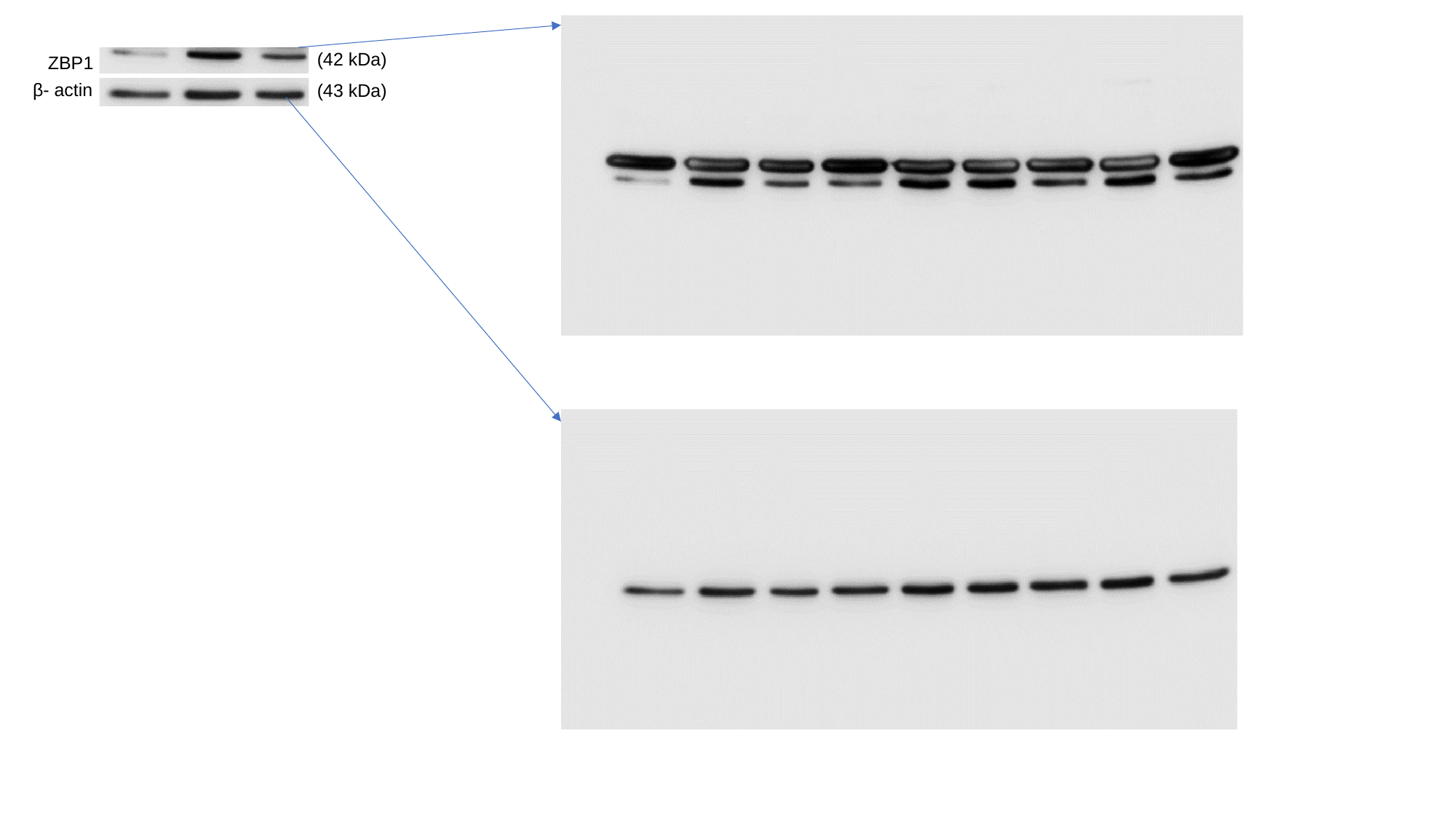

(42 kDa)
 ZBP1
β- actin
(43 kDa)
